# RNA Helicases in Microsatellite Repeat Expansion Disorders and Neurodegeneration

**DOI:** 10.3389/fgene.2022.886563

**Published:** 2022-05-12

**Authors:** Lydia M. Castelli, Bridget C. Benson, Wan-Ping Huang, Ya-Hui Lin, Guillaume M. Hautbergue

**Affiliations:** ^1^ Sheffield Institute for Translational Neuroscience (SITraN), Department of Neuroscience, University of Sheffield, Sheffield, United Kingdom; ^2^ Neuroscience Institute, University of Sheffield, Sheffield, United Kingdom; ^3^ Healthy Lifespan Institute (HELSI), University of Sheffield, Sheffield, United Kingdom

**Keywords:** microsatellite repeat expansion disorders, pathophysiology, RNA biology, repeat transcripts, polymeric repeat proteins, RNA helicases, therapeutic strategies

## Abstract

Short repeated sequences of 3−6 nucleotides are causing a growing number of over 50 microsatellite expansion disorders, which mainly present with neurodegenerative features. Although considered rare diseases in relation to the relatively low number of cases, these primarily adult-onset conditions, often debilitating and fatal in absence of a cure, collectively pose a large burden on healthcare systems in an ageing world population. The pathological mechanisms driving disease onset are complex implicating several non-exclusive mechanisms of neuronal injury linked to RNA and protein toxic gain- and loss- of functions. Adding to the complexity of pathogenesis, microsatellite repeat expansions are polymorphic and found in coding as well as in non-coding regions of genes. They form secondary and tertiary structures involving G-quadruplexes and atypical helices in repeated GC-rich sequences. Unwinding of these structures by RNA helicases plays multiple roles in the expression of genes including repeat-associated non-AUG (RAN) translation of polymeric-repeat proteins with aggregating and cytotoxic properties. Here, we will briefly review the pathogenic mechanisms mediated by microsatellite repeat expansions prior to focus on the RNA helicases eIF4A, DDX3X and DHX36 which act as modifiers of RAN translation in C9ORF72-linked amyotrophic lateral sclerosis/frontotemporal dementia (C9ORF72-ALS/FTD) and Fragile X-associated tremor/ataxia syndrome (FXTAS). We will further review the RNA helicases DDX5/17, DHX9, Dicer and UPF1 which play additional roles in the dysregulation of RNA metabolism in repeat expansion disorders. In addition, we will contrast these with the roles of other RNA helicases such as DDX19/20, senataxin and others which have been associated with neurodegeneration independently of microsatellite repeat expansions. Finally, we will discuss the challenges and potential opportunities that are associated with the targeting of RNA helicases for the development of future therapeutic approaches.

## Introduction

Microsatellite expansions have been attributed to numerous neurodegenerative diseases which are subdivided into either polyglutamine (poly-Q) and non-polyglutamine disorders. Poly-Q expansions encode a CAG trinucleotide repeat expansions and have been reported in Huntington disease (HD), Huntington disease-like 2 (HDL2), 7 subtypes of spinocerebellar ataxia (SCA 1-3,6,7,12,17), Dentatorubral-pallidoluysian atrophy (DRPLA), Spinal-Bulbar Muscular Atrophy (SBMA) and some Schizophrenia/migraines. Non-poly-Q expansion disorders encompass expansions of various lengths, ranging from trinucleotide to hexanucleotide repeats. Trinucleotide repeats include: 1) CGG repeats in Fragile X associated mental retardations; 2) CTG repeats in myotonic dystrophy (DM) and SCA8; 3) GAA repeats in Friedreich’s ataxia; 4) pentanucleotide (ATTCT, TGGAA) or hexanucleotide (GGCCTG) repeat expansions in spinocerebellar ataxia (SCA) type 10, 31, 36 respectively, and bi-directionally transcribed CTG/ CAG repeats in SCA8; 5) hexanucleotide (GGGGCC) repeat expansions in chromosome 9 open reading frame 72 (*C9ORF72*)-linked amyotrophic lateral sclerosis (ALS) and frontotemporal dementia (FTD). Several of these repeat expansions are bi-directionally transcribed including in Fragile X associated mental retardations, C9ORF72-ALS/FTD, HD, DM and several subtypes of spinocerebellar ataxia (SCA 2, 3, 8, 12, 31, 37). For recent reviews, we refer to (Castelli et al., 2021; Depienne and Mandel 2021).

### Pathophysiological Mechanisms Attributed to Microsatellite Expansions

Microsatellite expansions are located in coding and non-coding regions of the affected genes, with pathogenesis attributed to interconnecting mechanisms involving both RNA/proteins loss and gain of functions. Production of neurotoxic polymeric repeat proteins/peptides which are generated from repeat-associated non-AUG (RAN) translation in all frames and from coding or non-coding regions of genes were also evidenced in multiple repeat expansion disorders (reviewed in (Castelli et al., 2021; Depienne and Mandel 2021)).

Transcriptional silencing contributes to the pathophysiology caused by large expansions of a CGG trinucleotide (over 200) in the 5′-UTR region of the fragile X mental retardation 1 gene (*FMR1*), leading to the Fragile X syndrome (FXS), the most common inherited form of intellectual disability, due to haploinsufficiency and loss of the FMRP protein, which plays a role in repressing the translation of specific mRNAs for synaptic plasticity in dendrites. In contrast, the fragile X–associated tremor ataxia syndrome (FXTAS) is a late adult onset disease that involves intermediate expansions of 55–200 CGG repeats in the 5′-UTR of the *FMR1* gene. Unlike FXS, it leads to neurodegeneration and elevated levels of CGG-expanded *FMR1* transcripts in intranuclear neuronal and astrocyte inclusions (Tassone et al., 2004). The antisense repeat transcript spanning the 5′-UTR region of *FMR1* was also found to be up-regulated in individuals with intermediate CGG expansions (Ladd et al., 2007). The pathogenesis of FXTAS involve complex mechanisms including both RNA gain-of-functions by sequestration of RNA-binding factors (Sam68 (Sellier et al., 2010), PUR-alpha, hnRNP A2/B1, CUGBP1 (Jin et al., 2007; Sofola et al., 2007)) and protein gain-of-function by RAN translation of a poly-glycine-containing FMRP protein called FMRpolyG (Todd et al., 2013), which alter the ubiquitin proteasome system (Oh et al., 2015). For recent reviews on FXS/FXTAS, we refer to (Hagerman et al., 2017; Depienne and Mandel 2021).

Autosomal-dominant glutamine-encoding CAG repeat expansions (>36-250) in exon 1 of the Huntingtin gene (*HTT*) cause HD, while >41 CAG repeats in the 3′terminal exon of Junctophilin 3 (*JPH3*) are involved in HDL2. In addition, >32-88 CAG repeats lead to autosomal-dominant cerebellar ataxias (ADCAs) when inserted in the exonic regions of the following genes: 1) ataxin (*ATXN1, 2, 3 or 7*) in SCA types 1-3,7; 2) calcium voltage-gated channel subunit alpha1A (*CACNA1A*) in SCA6; 3) protein phosphatase 2 regulatory subunit B beta (*PPP2R2B*) in SCA12; 4) TATA-binding protein (*TBP*) in SCA17; 5) atrophin 1 (*ATN1*) in DRPLA; 6) X-linked androgen receptor (*AR*) in SBMA, 7) potassium calcium-activated channel subfamily N member 3 (*KCNN3*) in some Schizophrenia/migraines. In poly-Q disorders, the CAG repeat expansions are found in polymorphic regions encoding poly-glutamine stretches that lead to the translation of proteins with extended poly-Q domains that promote misfolding/ aggregation, reduced interactions with binding protein partners and abnormal interactions with other factors (toxicity through protein loss- and gain-of-functions). Moreover, RNA-mediated toxicity was also reported to directly contribute to pathogenesis of poly-Q disorders potentially through abnormal interactions with the muscleblind protein in *Drosophila* (Li et al., 2008) and nuclear retention (Tsoi et al., 2011; Tsoi and Chan 2013). In addition, it was also shown that RAN translation also occur through the coding CAG repeat expansion in the *HTT* alternative reading frames leading overall to both canonical translation of the HTT poly-Q expansion protein and to four additional RAN-translated sense and antisense homo-polymeric repeat proteins (poly-alanine, poly-serine, poly-leucine, poly-cysteine) that are toxic and aggregate in a CAG repeat length dependent mechanism in autopsied brains from human HD patients (Bañez-Coronel et al., 2015). For recent reviews on the pathological mechanisms involved in Poly-Q disorders, we refer to (Lieberman et al., 2019; Depienne and Mandel 2021).

In contrast to the poly-Q related SCAs, SCA8 was initially thought to be a novel form of disease caused by untranslated expansions of non-coding CTG repeats (>74) in the 3′-UTR of the *ATXN8OS* (*ATXN8* opposite strand) gene (Koob et al., 1999). The same group also found that antiparallel *ATXN8* transcripts with coding CAG repeats were also produced from the same locus encoding a poly-Q protein expressed in neuronal inclusions (Moseley et al., 2006). Moreover, RAN-translated poly-alanine proteins driven from *ATXN8* CAG repeat transcripts were further characterised in a mouse model of SCA8 and in human SCA8 brain tissue (Zu et al., 2011) suggesting that overall the pathogenesis of SCA8 involves both RNA (Daughters et al., 2009) and protein (Zu et al., 2011) toxic gain-of-functions. In human post mortem brains, the poly-alanine repeats proteins could also have been produced by RAN translation of the antiparallel *ATXN8OS* CTG repeat transcripts. For recent reviews on SCAs, we refer to (Klockgether et al., 2019; Depienne and Mandel 2021).

Repeat expansions of the CTG trinucleotide (50-10,000) in the non-coding 3′-UTR of the myotonic dystrophy protein kinase gene (*DMPK*) and of CCTG repeats (75-1,100) in intron 1 of the Zinc Finger Protein 9 gene (*ZNF9*) lead to myotonic dystrophy (DM1) and myotonic dystrophy type 2 (DM2) respectively. In DM1, these expansions are thought to predominantly cause RNA-mediated toxicity through loss-of-function of regulatory RNA-binding/splicing factors (including the muscleblind-like splicing regulator (MBNL) and CUG binding protein and ETR3-like factor (CELF) families of proteins) sequestered on hairpin structures formed by the CUG repeat transcripts that form characteristic intranuclear RNA foci. In addition, RAN translation of various repeat proteins were evidenced in DM1 and DM2 post-mortem brains as well as *in vitro* (Zu et al., 2011; Zu et al., 2017). For recent reviews on myotonic dystrophies, we refer to (Meola and Cardani 2015; Depienne and Mandel 2021).

Hexanucleotide-repeat GGGGCC expansions (30-4,400) in the first intron of the *C9ORF72* gene are the most commonly identified genetic cause of amyotrophic lateral sclerosis (ALS) and frontotemporal dementia (FTD) (DeJesus-Hernandez et al., 2011; Renton et al., 2011). Bi-directional transcription of sense and antisense repeat transcripts trigger neuronal injury through multiple non-mutually exclusive mechanisms. A large body of evidence in cell and animal models implicate RNA/protein toxic gain-of-functions through repeat-RNA sequestration of SRSF1, which triggers the nuclear export of *C9ORF7*2-repeat transcripts retaining pathological repeat expansions in intron-1 (Hautbergue et al., 2017), and subsequent RAN translation of five sense and antisense dipeptide-repeat proteins (DPRs) (poly-glycine-alanine, poly-glycine-arginine, poly-glycine-proline, poly-proline-alanine and poly-proline-arginine dipeptide containing proteins) (Mori et al., 2013; Ash et al., 2013; Zu et al., 2013) as one on the main drivers of neurotoxicity. Additional pathological mechanisms which may contribute to disease progression/severity involves protein loss-of-functions through sequestration of RNA-processing factors onto nuclear/cytoplasmic sense and antisense RNA foci as well as haploinsufficiency due to decreased expression levels of the C9ORF72 protein. For recent reviews on the *C9ORF72*-repeat mediated pathological mechanisms, we refer to (Balendra and Isaacs 2018; Gendron and Petrucelli 2018; Jiang and Ravits 2019; McEachin et al., 2020; Yang et al., 2020; Depienne and Mandel 2021).

### RNA Helicases in Canonical Translation

**FIGURE 1:**
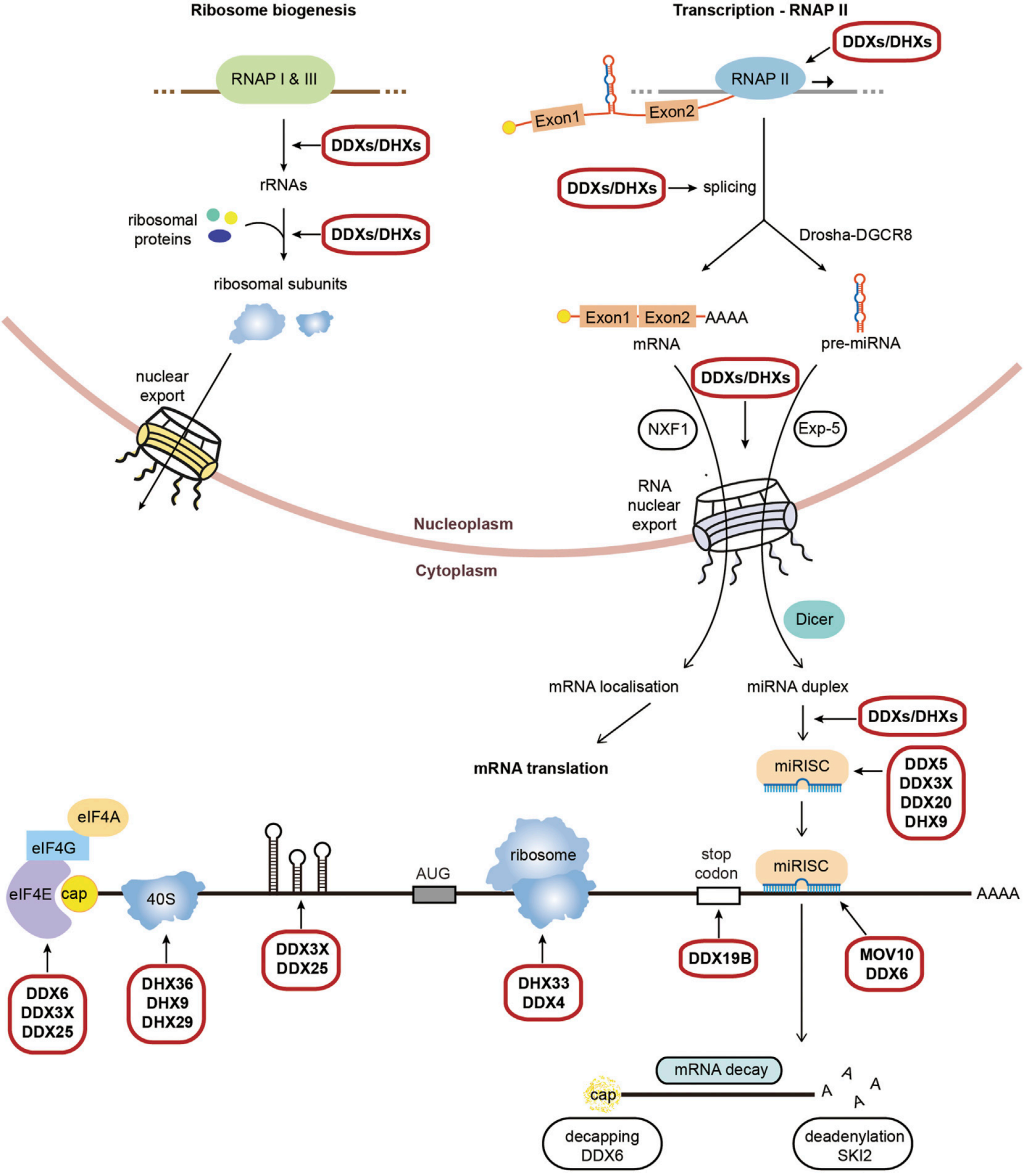
Physiological roles of RNA helicases in mammalian gene expression. Schematic representation of RNA helicases (DDXs/ DHXs) which function across RNA metabolic processes from transcription to translation and decay. Highlighted are the processes which require RNA helicases to unwind secondary structures, with an emphasis on the process of translation. We refer to Supplementary Table S1 for a detailed list and roles of RNA helicases in the different steps of gene expression.

RNA helicases are motor proteins that catalyse the ATP-dependent unwinding of RNA duplexes in a wide array of RNA metabolic processes, from pre-mRNA splicing, RNA nuclear export, ribosome biogenesis up to translation and mRNA decay/degradation, with a large number of RNA helicases functioning in multiple steps (Figure 1). They play a vital role in sensing viral RNA, facilitating antiviral immune responses and are essential for viral replication, either through viral RNA helicases or hijacking cellular RNA helicases (Steimer and Klostermeier 2012). RNA helicases are grouped into 2 superfamilies SF1 and SF2, although the majority belongs to the DExH and DEAD box families within SF2 (Bourgeois et al., 2016). A detailed list of RNA helicases and their roles in the mammalian expression of genes is provided in Supplementary Table 1. RNA helicases have moreover been linked with cancers, infectious diseases and several neurodegenerative disorders, including ALS, spinal muscular atrophy, spinocerebellar ataxia and Alzheimer’s disease.

RNA helicases are involved at multiple steps of translation, however most function at the initiation step of translation. Once exported to the cytoplasm the cap-binding complex (CBC) of capped mRNA is replaced by the cap binding protein eIF4E, which interacts with eIF4G and eIF4A to form the eIF4F complex. The RNA helicase eIF4A comprises two isoforms, eIF4A1 (also known as DDX2A) and eIF4A2 (also known as DDX2B), which actively unwinds secondary structures within 5′-UTRs along with its cofactors eIF4B and eIF4H (Svitkin et al., 2001; Pestova and Kolupaeva 2002; Wolfe et al., 2014). Other RNA helicases have been implicated in the unwinding the 5′-UTR containing secondary structure of specific subsets of mRNA, including DDX3 which regulates transcripts including RAC1 or cyclin E1 (Lai et al., 2010; Soto-Rifo et al., 2012; Chen et al., 2015), DDX6 which enhances the translation of a subset mRNA involved in self-renewal and proliferation (Y. Wang et al., 2015) and DDX25 which controls expression of mRNA involved in spermatogenesis (Tsai-Morris et al., 2012). DDX3X, an isoform of DDX3, also functions in other translational control mechanisms, including the inhibition of Drosha-mediated processing of a subset of pri-miRNAs (Krol et al., 2015) and the facilitation of miRISC formation (Pek and Kai 2011; Poulton et al., 2011; Kasim et al., 2013).

Other RNA helicases facilitate or repress translation through resolving specific secondary structures such as G4 quadruplexes (G4s). These include DHX9, DHX36, DDX5, DDX17 and MOV10L1 facilitate the translation of specific mRNAs which harbour G4s within their 5′-UTR (Murat et al., 2018; Chen et al., 2021; Dardenne et al., 2014; Vourekas et al., 2015). DHX9 (RHA) also facilitate 40S scanning and ribosome recycling or mRNA circularisation (Peng et al., 2011; Manojlovic and Stefanovic 2012; Halaby et al., 2015); while DHX36 (RHAU) enhances deadenylation and decay of transcripts with 3′-UTR AU rich elements through PARN complex (Tran et al., 2004), the release of a subset of mRNAs from stress granules for translation (Chalupníková et al., 2008) and competes with Dicer to inhibit the maturation of miRNA-134 (Bicker et al., 2013). Interestingly, DHX36 is involved in both the translation and degradation of the Nkx2-5 mRNA via interactions with both 5′-UTR and 3′-UTR G4s (Nie et al., 2015). DHX29 directly binds the 40S ribosome subunit to stimulate initiation by ensuring the correct positioning of the ribosome entry channel (Pisareva et al., 2008; Dhote et al., 2012) while DHX33 was reported to promote assembly of the 80S ribosome at late stage of translation initiation (Zhang et al., 2015).

Other RNA helicases are recruited to the 3′-UTRs and are involved in translation control and termination. DDX19B (DBP5) facilitates stop codon recognition and recruitment of eukaryotic Release Factor eRF3, the polypeptide chain release factor to promote terminating complexes but also plays additional roles with eukaryotic elongation factors eEF1/eEF2 in stabilizing ribosomal elongation complexes (Mikhailova et al., 2017); while DHX30 (Rizzotto et al., 2020; Bosco et al., 2021) and DDX21 regulates distinct subset of mRNAs via G4s present in 3′-UTRs (McRae et al., 2020). DDX21 has also been shown to repress other transcripts via G4s in the 5′-UTR (McRae et al., 2017). DDX6 (RCK) enhances the translation of a subset of proliferation mRNAs in epidermal progenitor cells (Y. Wang et al., 2015) and facilitates the interplay between miRNA mediated post-transcriptional gene silencing (Roy and Jacobson 2013; Huch and Nissan 2014). DDX6 additionally stabilises autophagy mRNAs through association with DCP2 (Hu et al., 2015) and enhances 5′-3′ mRNA decay by facilitating XRN1 access for decapping (Coller et al., 2001; Fischer and Weis 2002). A further helicase, SKI2, facilitates 3′-5′ deadenylation and mRNA decay (Johnson and Jackson 2013).

Interconnected with translation is miRNA gene silencing of translation and several RNA helicases function in both processes. miRNAs are short non-coding RNA sequences which form miRNA-induced silencing complexes (miRISCs) with Argonaut proteins and mediate post-transcriptional gene silencing (Ha and Kim 2014; Jonas and Izaurralde 2015). Silencing occurs through either translation inhibition or mRNA degradation (Jonas and Izaurralde 2015), with translational repression facilitated by eIF4A2 recruitment to the 5′-UTR (Meijer et al., 2013). Many RNA helicases facilitate miRNA biogenesis and miRISC assembly on target mRNAs. In the nucleus, DDX5 and DDX17 are both required for the maturation of pre-miRNAs (Moy et al., 2014; Motiño et al., 2015), while DHX9 (Kawai and Amano 2012), DDX1 (Gregory et al., 2004; Han et al., 2014), DDX23 (Yin et al., 2015) and DDX25 (Dai et al., 2011) are involved in the subsequent processing of the pri-miRNAs. Following export, Dicer-cleaved pre-miRNAs are cleaved further processed by RNA helicases including DDX5 (Ulvila et al., 2006), DDX20 (Takata et al., 2012), DHX9 (Robb and Rana 2007) and MOV10 (Kenny et al., 2014), to promote formation of the miRISC silencing complexes.

## RNA Helicases Associated With Microsatellite Repeat Expansion-Mediated Neurodegeneration

### The Eukaryotic Initiation Factor eIF4A Resolves RNA Secondary Structures to Initiate RAN Translation

The eukaryotic Initiation Factors eIF4A (eIF4A1, eIF4A2 and eIF4A3) are highly conserved family of RNA helicases, functioning in the unwinding of RNA secondary structures, catalysation of RNA annealing and displacement of proteins from RNA. While eIF4A1 and eIF4A2 are involved in the control of translation, eIF4A3 is a subunit of the exon junction complex which stimulate mRNA nuclear export as well as translation but is also involved in inducing nonsense mediated mRNA decay (NMD) of transcripts with premature stop codons. They form large, multi-protein complexes to fulfil these tasks, interacting through their N and C-terminal domains. While research attention has predominately come from the field of cancer biology, helicases are now understood to also play an important role in neurodegenerative diseases. In both canonical and RAN translation, the RNA helicases eIF4A unwind and facilitate the scanning of mRNA for a start codon, with translation proceeding upon recognition of an AUG in the case of canonical translation and in some cases a near-cognate start codon (reviewed in (Castelli et al., 2021)). An ever-increasing number of rocaglate inhibitors of eIF4A activity have been identified to date which target either helicase or mRNA binding activity (Cruz-Migoni et al., 2011; Tillotson et al., 2017; Abdelkrim et al., 2018) and several of these have been utilized to study the role of eIF4A in canonical and non-canonical translation. The inhibitor hippuristanol was used to highlight the role of eIF4A in the translation of two proteins which have a major contributory role in AD which have long structured 5′-UTRs such as in the microtubule associated protein Tau (*MAPT*) and amyloid β precursor protein (*APP*) genes encoding Tau and amyloid-beta peptide (Bottley et al., 2010). Interestingly, this study showed that translation of neuroprotective proteins with short 5′-UTRs such as SOD1, TXN and NDUFB2 were unaffected by hippuristanol treatment (Bottley et al., 2010).

Transient secondary structures forming in mRNA transcripts are important in regulatory control of canonical translation, but the *C9ORF72*-repeat expansions are particularly prone to forming highly stable G-quadruplex structures due to their G-rich nature (Fratta et al., 2012). G-quadruplexes are comprised of stacked guanine tetrads, joined by lateral loops and stabilised by hydrogen bonding (Rhodes and Lipps 2015; Mendoza et al., 2016). They are dynamic structures and their important regulatory function in the fine control of gene expression is reflected in the fact that they are observed throughout the human genome. In particular, they are found in regions including promoters, splice sites and 5′-UTRs (Chambers et al., 2015). eIF4A and other RNA helicases resolve secondary structures in an ATP-dependent manner using a crank-like mechanism, one base at a time, thereby enabling ribosomal scanning and RAN translation to proceed. The action of helicases are therefore particularly important in the context of this, and other, neurodegenerative diseases caused by microsatellite repeat expansion. The eIF4A inhibitor hippuristanol significantly reduced RAN translation of CGG-repeat expansions in *FMR1* in FXTAS (Kearse et al., 2016) and GGGGCC-repeat expansions in C9ORF72-ALS/FTD (Green et al., 2017). Use of eIF4A-specific inhibitor FL3 further confirmed a role of eIF4A in reducing C9ORF72 DPR production in HEK293 cell models without decreasing levels of repeat-containing mRNA transcripts (Tabet et al., 2018), providing clear evidence for the requirement of eIF4A in the initiation of RAN translation. Interestingly, the depletion of eIF4B and eIF4H, co-factors which stimulate the activity of eIF4A, suppresses the neurodegenerative-associated rough eye phenotype of *Drosophila* models of FXTAS (Linsalata et al., 2019) and C9ORF72-ALS/FTD (Goodman et al., 2019), while the down-regulation of eIF4A either show no rescue effect or is lethal depending on the level of depletion.

On the other hand, independently of its role in RAN translation, eIF4A1 promotes the unwinding of non-Watson-Crick RNA:RNA intermolecular interactions which occur during condensation of RNA molecules in stress granules, leading to reduced formation of stress granules which have been implicated in pathological mechanisms induced by *C9ORF72*-repeat expansions (Tauber et al., 2020).

### DEAD-Box Helicase DDX3X Functions in RAN Translation

DDX3X is an ATP-dependent RNA helicase which functions in multiple steps of RNA metabolism both within the nucleus and the cytoplasm (Soto-Rifo and Ohlmann 2013). DDX3X plays a crucial role in determining cell fate after stress, with pro-survival stress granules and programmed cell death NLRP3 inflammasomes competing for DDX3X binding (Samir et al., 2019). Defective assembly and disassembly of stress granules has been implicated in disease, including neurodegeneration (Wolozin 2012) and DDX3X mutation or dysregulation has been linked to several neurodegenerative and neurodevelopmental disorders (Snijders Blok et al., 2015; Kellaris et al., 2018; Lennox et al., 2020). Similar to eIF4A, DDX3X enhances the translation of RNAs containing strong secondary structures such as IRES (Su et al., 2018) or G-quadruplexes (Herdy et al., 2018). And a range of inhibitors are being developed as anti-viral or anticancer treatments (Cui et al., 2020; Kukhanova et al., 2020).

Interestingly, more recent studies have implicated a role for DDX3X in RAN translation of microsatellite repeats. DDX3X was found to bind to the GC-rich 5′-UTR of *FMR1* containing CGG repeats, although this appeared independent of the CGG repeat (Linsalata et al., 2019) and *C9ORF72* GGGGCC_40_ repeats but not CCCCGG_40_ repeats (Cheng et al., 2019). Binding of DDX3X to GGGGCC_40_ was independent of its ATPase activity and was not due to direct binding G4-quadruplex structures (Cheng et al., 2019). Interestingly, DDX3X binds both partial dsRNA duplexes (Epling et al., 2015) and G4-structures (Herdy et al., 2018) and the *C9ORF72* GGGGCC repeats adopts a mixture of hairpin and G4-structures (Su et al., 2014) suggesting DDX3X interacts with specific hairpin structures within *C9ORF72*-repeat expansions and the 5′-UTR of *FMR1*. DDX3X binding of the 5′-UTR of *FMR1* facilitates the RAN translation of the FMRpolyG RAN product, with suppression of DDX3X reducing translation of FXTAS CGG-repeats and recuing the associated toxicity in *Drosophila*, a HeLa reporter system and primary neurons (Linsalata et al., 2019). However, this role in facilitating RAN translation if sequence specific, with the opposite observed for *C9ORF72* repeat-driven RAN translation (Cheng et al., 2019). A suppression of DDX3X led to an increase in RAN translation in a HeLa reporter system, *Drosophila* model and a range of patient-derived cell lines, while overexpression of DDX3X suppressed RAN translation in C9ORF72-ALS/FTD (Cheng et al., 2019). DDX3X depletion or overexpression had no effect on the expression of antisense polyPA and polyPR DPRs, in agreement with it not binding the antisense CCCCGG-repeat expansions (Cheng et al., 2019). Depletion of DDX3X also appeared to impact translation, with no effect observed on nuclear export of the repeat expansions, stress granule formation, apoptosis, cell proliferation or activation of the Integrated Stress Response (ISR) (Cheng et al., 2019). One hypothesis to explain the roles of DDX3X in the stimulation (Linsalata et al., 2019) or inhibition (Cheng et al., 2019) of RAN translation in FXTAS or C9oRF72-ALS/FTD respectively would be to consider the secondary structures of the repeat RNAs, with the CGG-repeats adopting predominantly G-quadruplexes while sense GGGGCC-repeats form a mixture of G-quadruplexes and hairpins. Cheng et al., 2019 suggested that DDX3X would preferentially bind hairpins structures in an ATP-binding and RNA-helicase independent manner which leads to reduced unwinding and RAN translation of sense *C9ORF72*-repeat transcripts. Another study indicated the physiological relevance of the RNA hairpin structures formed by the sense *C9ORF72*-repeats in cell models through the identification of small molecule inhibitors selectively targeting the hairpin structures (Wang et al., 2019).

### DEAH-Box Helicase DHX36 Functions in RAN Translation

DHX36 has been described as the most important resolver of secondary structures *in vivo,* responsible for resolving the majority of G-quadruplexes in the genome and binding to a diverse range of secondary structures with very high affinity (Vaughn et al., 2005). Similar to DDX3X, DHX36 functions in multiple steps of RNA metabolism and has been reported to interact with G-rich sequences prone to G-quadruplex formation in over 4,500 mRNA transcripts. DHX36 is important in the unwinding of G4 quadruplexes within telomeres, with DHX36 depletion leading to reduced telomere length (Booy et al., 2012; Booy et al., 2015). DHX36 knockout is associated with increased mRNA transcript abundance but decreased protein output, highlighting its crucial role in translation (Sauer et al., 2019). Such a role has been demonstrated for the G-quadruplex (GGN)_13_ repeat with the 5′-UTR of the potassium 2 pore domain leak channel protein Task3, enabling its translation (Maltby et al., 2020), along with several mRNAs essential for skeletal muscle regeneration (X. Chen et al., 2021).

In the context of RAN translation, DHX36 has been shown to bind the *C9ORF72*-repeat G4-quadruplexes with high affinity (Liu et al., 2021; Tseng et al., 2021) and unwind the secondary structure by hydrolyzing ATP (Liu et al., 2021). DHX36 facilitates RAN translation of the *C9ORF72*-repeat transcripts, with stable depletion of DHX36 suppressing RAN translation of HeLa cells expressing a polyGP C9-RAN_70_ reporter system either stably (Liu et al., 2021) or a C9-RAN_70_ reporter system transiently in all three reading (Tseng et al., 2021). Stable knockout of DHX36 in Jurkat cells replicated this suppression, in a repeat length dependent manner; while overexpression of DHX36 increased RAN translation through its helicase domain (Tseng et al., 2021). Additionally, stable knockdown of DHX36 in C9ORF72-ALS patient derived iPSC cells and induced motor neurons also showed a reduction in the polyGP RAN product and DHX36 protein levels were found to be elevated in C9ORF72-ALS spinal cord tissue (Liu et al., 2021). The role of DHX36 in RAN translation was further confirmed using a FMRpolyG reporter construct expressing 100 CGG repeats, with knockdown of DHX36 impeding RAN translation (Tseng et al., 2021). DHX36 has additionally been hypothesised to impact on translation elongation, with depletion therefore linked to ribosomal stalling and slower translation (Liu et al., 2021; Tseng et al., 2021) and indeed DHX36 has been implicated in facilitating translation elongation of G4-containing mRNAs (Endoh et al., 2013). Interestingly, DHX36 may impact on microsatellite repeat expansions at multiple steps of RNA metabolism as it does with canonical transcripts. DHX36 was shown to enhance transcription of C9-RAN reporter constructs (Tseng et al., 2021) and plays an integral role in stress granule formation, G4-induced cellular stresses and the integrated stress response (ISR) (Chalupníková et al., 2008; Byrd et al., 2016; Sauer et al., 2019). The ISR is activated during RAN translation of both *C9ORF72* and *FMR1* repeat expansions and has been shown enhance RAN translation in a feedback loop (Green et al., 2017; Cheng et al., 2018; Sonobe et al., 2018; Westergard et al., 2019) and interestingly depletion of DHX36 was shown to prevent this enhancement following activation of the ISR (Tseng et al., 2021). Overall, further work into the role of helicases in stress and the link with RAN translation across repeat expansion disorders remains to be established.

### Dysregulation of Nuclear RNA Metabolic Processes in Microsatellite Expansion Disorders

Several RNA helicases facilitate splicing and alternative splicing of mRNAs (reviewed in (Bourgeois et al., 2016)) and dysregulation of splicing has been reported for microsatellite repeat expansion disorders, including DM, C9ORF72-ALS/FTD and several Spinocerebellar ataxias (reviewed in (Hale et al., 2019)). RNA-mediated toxicity due to aberrant splicing events is the main driver for CUG-expanded repeats in DMPK degeneration in DM (reviewed in (Pettersson et al., 2015)). This dysregulation of splicing has been shown to be regulated by the proteins MBNL1 and CUGBP1 (Ho et al., 2004; Ho et al., 2005) with over 80% of the splicing defect attributed to a loss of function of MBNL1 (Du et al., 2010). The DEAD-Box Helicases DDX5 (p68) and DDX17 (p72) along with the DEAH-Box helicase DHX9 were all shown to bind MBNL1 with greater affinity in DM1, along with other non-helicase interacting proteins involved in splicing (Paul et al., 2011). DDX5, DDX17 and DHX9 all had elevated levels in myoblasts from DM1 patients and resulted in an altered MBNL1 stoichiometry in DM myoblasts (Paul et al., 2011). This increase in protein levels was due to translational upregulation and not post-translational modifications (Paul et al., 2011). Furthermore, DDX17 and DDX5 were shown to form complexes on the CUG repeat expansions and colocalised with RNA foci, a hallmark of DM1 (Laurent et al., 2012). They were shown in facilitate binding of MBNL1 to the repeat expansions in a helicase dependent manner, suggesting that they unwind the secondary structure of the repeat RNA, increasing its affinity for MBNL1 (Laurent et al., 2012). Depletion of DDX5 was found to modestly reduce the nuclear CUG-foci (Laurent et al., 2012). Interestingly, DDX17 and DDX5 shares over 90% homology of the helicase core domain with DDX5, however they have divergent N and C terminal domains, and are unable to compensate for one another past the very early stages of development (Giraud et al., 2018). Furthermore, DDX5 and DDX17 dysregulation has also been linked to the *C9ORF72*-repeat expansions through interactions with polyPR aggregates (Suzuki et al., 2018). Both DDX5 and DDX17, along with DDX18, interact with polyPR aggregates in an RNA-dependent manner (Suzuki et al., 2018) resulting in a reduction in ribosome biogenesis (Suzuki et al., 2018). Furthermore, polyPR was shown to directly inhibit the function of DDX5 (Suzuki et al., 2018).

Another RNA helicase shown to modulate DM1 pathology is DDX6, which appears to regulate the homeostasis of nuclear foci in DM1, countering the effects of DDX5 and DDX17 (Pettersson et al., 2014). Depletion of DDX6 was shown to increase the number and intensity of CUG nuclear foci, while overexpression of DDX6 reduced the number of nuclear foci (Pettersson et al., 2014). The reduction in nuclear foci additionally resulted in small cytoplasmic foci which did not colocalise with processing body markers (Pettersson et al., 2014). Displacement of MBNL1 from the CUG repeats and a partial relief of the DM1 splicing defect in IR2 mRNA was also observed following overexpression of DDX6 (Pettersson et al., 2014). Taken together, it appears the predominantly cytoplasmic DDX6 binds the CUG repeats in the nucleus and displace MBNL1 in an ATP dependent manner, mediating the transition between CUG repeat foci and diffuse CUG repeat mRNP complexes and countering the splicing defects facilitated by DDX5 and DDX17 (Pettersson et al., 2014).

It is also notable that DDX17 has been identified as a modifier of FUS-driven neurodegeneration (Fortuna et al., 2021). Mutations in the RGG1 domain of the *FUS* gene recruit nuclear DDX17 into cytoplasmic stress granules disrupting its regulation of the DNA damage pathway (Fortuna et al., 2021). *Drosophila* models and iPSC cells from FUS-ALS patients all exhibit reduced levels of DDX17 and overexpression of DDX17 upregulated the DNA damage machinery, rescuing FUS-mediated toxicity (Fortuna et al., 2021). Interestingly, a *Drosophila* study of DDX17 orthologs, DDX3 and DDX4 which contain a similar RGG motif to DDX17 were also found to also modulate the neurodegenerative phenotype induced by mutant FUS, providing further evidence for the vital role of the RGG motif as underlying DDX17 and FUS interactions (Fortuna et al., 2021).

### Dysregulation of Dicer and the RNA Interference Pathways in Microsatellite Expansion Disorders

Dicer is an essential RNA helicase which maintains cellular physiology and is integral to the RNA interference pathways. Dicer is a large 220 kDa protein which is located within the cytoplasm and fragments long dsRNAs or pre-miRNAs into short dsRNAs or miRNAs respectively. Dysregulation of Dicer expression levels are implicated in many human diseases, with both up- or down-regulation of Dicer linked to neurodegeneration, including repeat-associated disorders. The expression level of Dicer has been shown to decrease with age, linking it to age-related neurodegeneration (Mori et al., 2012; Yan et al., 2013). Downregulation of Dicer has been implicated in neurodegenerative diseases including neuropsychiatric disorders such as chronic stress and depression (Dias et al., 2014; Wingo et al., 2015), multiple sclerosis (MS) (Aung and Balashov 2015; Magner et al., 2016) and Parkinson’s Disease (PD) (Simunovic et al., 2010). Depletion of Dicer in dopaminergic neurons mimics PD (Chmielarz et al., 2017) and expression of the Dicer stimulant enoxacin was neuroprotective in PD models (Chmielarz et al., 2017). Moreover, a loss of Dicer in motor neurons leads to progressive motor neuron degeneration and ALS (Haramati et al., 2010; Chen and Wichterle 2012).

Dicer also functions in pathogenesis from triplet repeat expansion disorders, including DM1, HD, FXTAS and SCA1, where it cleaves the triplet repeat dsRNA hairpin structures into short (∼21 nucleotide) CNG dsRNAs (Krol et al., 2007; Bañez-Coronel et al., 2012; Kelley et al., 2012). The generation of these short antisense CNG repeats results in siRNAs which can function in downregulating their long precursor repeat transcripts (Krol et al., 2007; Llamusí and Artero 2008). However, the generation of these short RNAs is also correlated with a reduced neuronal viability and are present in elevated levels in brains from HD patients compared to healthy controls; with anti-miRNAs to the short CAG repeats of HD relieving HD associated neurotoxicity (Bañez-Coronel et al., 2012). Furthermore, the toxic production of short RNA repeats by Dicer in fibroblasts from DM1 (siCUG) and HD (siCAG) has also been reported (Krol et al., 2007). The presence of cytoplasmic *C9ORF72*-repeat dsRNA in C9ORF72-ALS/FTD post-mortem brains was also recently reported to induce the type I interferon (IFN-I) inflammatory response and neuronal death in neural cells and mice, implicating further pathological activation of the atypical DExD/H-box RNA helicase MDA5 (Rodriguez et al., 2021). It is also worth noting that miRNA dysregulation has further been linked with the *C9ORF72*-repeat driven neurodegeneration (Porta et al., 2015; Varcianna et al., 2019; Kmetzsch et al., 2021). The precise mechanisms of how this pathway is dysregulated remain to be elucidated, however RNA helicases, with functions in other RNA metabolic processes, such as DDX5 and DDX3X have been shown to be disrupted (Suzuki et al., 2018; Cheng et al., 2019). Further studies to investigate the effect of these helicases on RNA interference pathways are required.

### Dysregulation of the UPF1 and the Nonsense-Mediated mRNA Decay Pathway in Microsatellite Expansions Disorders

The RNA helicase UPF1 is an essential component of the mRNA surveillance pathway responsible for sensing and degrading aberrant mRNA transcripts, e.g., mRNAs harbouring premature stop codons (PTCs), termed the nonsense mediated mRNA decay (NMD) pathway. UPF1 specifically enables the processive unwinding NMD target mRNAs, remodelling the mRNP complexes present on the mRNAs and facilitating their degradation by the NMD machinery (Fiorini et al., 2015). UPF1 expression levels are tightly controlled during neural differentiation, with a reduction of UPF1 essential for differentiation to occur (Lou et al., 2014). This regulation of UPF1 levels is mediated by the miRNA pathway with miR128 repressing translation of UPF1 during differentiation (Bruno et al., 2011; Lou et al., 2014).

Dysregulation of UPF1 has been implicated in *C9ORF72*-repeat driven neurodegeneration. The DPRs polyGR and polyPR have been shown to inhibit UPF1 mediated decay, including NMD (Xu et al., 2019; Ortega et al., 2020; Sun et al., 2020). Ortega et al. (2020) identified *C9ORF72*-repeat transcripts as a target of NMD via accumulation of eRF1 in specific nuclear envelope invaginations that confer a neuroprotective effect by mediating the transition from translation to NMD-dependent mRNA decay. The polyGR and polyPR proteins also recruit UPF1 to stress granules in an NMD independent manner (Xu et al., 2019; Sun et al., 2020) and reduce the presence of processing bodies (Xu et al., 2019). The dysregulation of UPF1 by these arginine containing DPRs is postulated to contribute to the *C9ORF72*-repeat driven pathology by preventing NMD to occur and indeed overexpression of UPF1 rescues the neurotoxicity observed in disease models (Xu et al., 2019; Ortega et al., 2020; Sun et al., 2020; Zaepfel et al., 2021). In support of this, neuroprotection was also observed pharmacologically with the addition of the NMD upregulating drug tranilast (Xu et al., 2019). However, upregulation of other NMD components did not rescue the toxicity (Sun et al., 2020)and the overexpression of UPF1 has been shown to rescue neurotoxicity without altering the NMD pathway (Sun et al., 2020; Zaepfel et al., 2021) and indeed the NMD defect resulting from these DPRs appears to be from a reduction in global translation rather than the alterations to the pathway (Sun et al., 2020). Furthermore, overexpression of UPF1 reduces the abundance of polyGP DPR levels, with depletion of UPF1 increasing the levels, in an NMD independent manner highlighting involvement of UPF1 in another, yet unidentified, pathway linked to neurotoxicity (Zaepfel et al., 2021). Interestingly, UPF1, along with other members of the quality control machinery, aggregates with mutant Huntington into immobile inclusions (Ormsby et al., 2020) however overexpression of UPF1 did not confer neuroprotection in HD models as has been observed for C9ORF72-ALS/FTD (Barmada et al., 2015).

Dysregulation of UPF1 has additionally been linked to the loss of function of FMRP1 due to the CGG repeats which result in FXTAS. A loss of the FMRP1 protein results in a hyperactive NMD pathway (Kurosaki et al., 2014). UPF1 binds FMRP1 directly, promoting its binding to NMD targets and preventing NMD, therefore a loss of the FMRP1 protein results in increased NMD (Kurosaki et al., 2014). The FMRP1 protein also binds another RNA helicase involved in RNA interference pathways, MOV10, protecting a subset of transcripts from AGO2-mediated decay (Kenny et al., 2014; Kenny et al., 2020). Clearly the loss of function of FMRP1 results in dysregulation of multiple RNA decay pathways. Furthermore, an NMD independent role for UPF1 has been identified through its interactions with STAU2 (Graber et al., 2017). UPF1 and STAU2 mediate the transport of RNA granules and local translation of proteins essential for neural plasticity which is deregulated in FXTAS (Graber et al., 2017). Further work is required to elucidate how this NMD independent function of UPF1 impacts on FXTAS.

**FIGURE 2:**
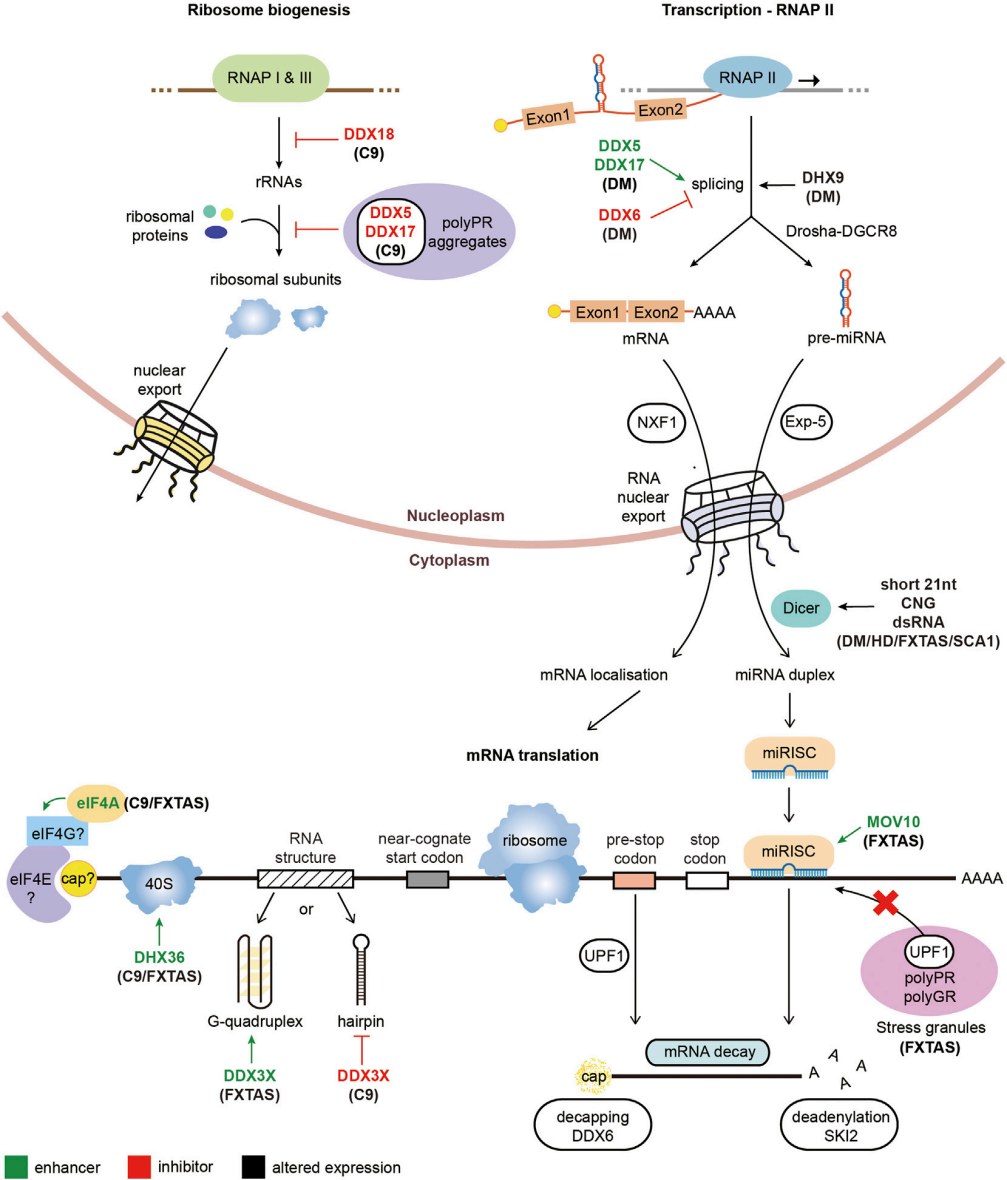
Pathological roles of RNA helicases in microsatellite expansion disorders. Diagrammatic representation of RNA metabolic functions with RNA helicases implicated in repeat expansion diseases. RNA helicases labeled in green enhance the arrow-indicated processes while RNA helicases typed in red inhibit the corresponding highlighted step of gene expression. On the other hand, RNA helicases with black text have altered expression levels in the associated repeat expansion disorders.

The overexpression of UPF1 has been shown to ameliorate the neurotoxicity associated with other genetic causes of ALS. ALS mutations with the *FUS* gene have been shown to alter the stoichiometry of proteins involved in NMD, including UPF1, highlighting manipulation of this pathway as a potential therapeutic target for FUS-ALS (Kamelgarn et al., 2018). Indeed, overexpression of UPF1 in FUS-ALS models suppresses the observed neurotoxicity (Ju et al., 2011; Barmada et al., 2015), however overexpression of UPF1 did not reduce the presence of FUS inclusions, bringing into question whether these inclusions exhibit a toxic effect (Ju et al., 2011). Additionally, overexpression of UPF1 was shown to rescue neurotoxicity in TDP43-ALS models (Jackson et al., 2015; Barmada et al., 2015). However, UPF1 overexpression does not alleviate all genetic causes of ALS, with SOD1-ALS models unaffected by increased UPF1 levels (Barmada et al., 2015). The neuroprotection observed by UPF1 appeared to be through modulation of the NMD pathway for FUS-ALS and TDP43-ALS, as pharmacological inhibition of NMD increased the observed neurotoxicity (Barmada et al., 2015). A summary diagram of the RNA helicases involved in regulating rRNA, ribosome or miRNA biogenesis, splicing and RAN translation in repeat expansion disorders is represented in Figure 2.

### RNA Helicases Associated With Non-Repeat Associated Neurodegeneration

Dysregulation of RNA helicases have been implicated in other neurodegenerative disorders including other genetic causes of ALS, PD, AD, intellectual disabilities and neurodegeneration linked to genome instability. Over 20 genetic causes of ALS have been identified and several of these result in dysregulation of RNA metabolism. As mentioned previously, upregulation of UPF1 rescues the neurotoxicity of FUS-ALS and TDP43-ALS implicating this RNA helicase in the disease aetiology of these mutated genes. However, UPF1 is not the only RNA helicase implicated in these diseases. DDX20 (Gemin3) is involved in multiple processes, however its most documented role is as a core member of the essential multiprotein survival of motor neurons (SMN) complex, which is involved in snRNP assembly during splicing (Charroux et al., 1999). SMN is the disease determining gene in the childhood neurodegenerative disease spinal muscular atrophy (SMA). Genetic association analysis has highlighted that DDX20 functionally interacts with TDP43 and FUS in pathways associated with viability, muscle mass, motor ability and neuromuscular junctions (Cacciottolo et al., 2019). Furthermore, DDX20 has been shown to be indispensable for the survival of neural progenitor cells and oligodendrocyte progenitor cells highlighting its role in neuronal processes (Simankova et al., 2021; Bizen et al., 2022). Additionally, the RNA helicase DDX58 (Rig-1) has also been shown to be misregulated in TDP43-ALS (MacNair et al., 2016); while genome wide analysis has highlighted DHX58 and other components of the TBK1-related immune pathway as a risk factor for the form of FTLD-TDP (Pottier et al., 2019). Interestingly, FTD exists on a disease spectrum with ALS and mutations in TBK1 have been identified as causative of both ALS and FTD (Cirulli et al., 2015; Freischmidt et al., 2015; Pottier et al., 2015) highlighting the potential role of DHX58 in these diseases. Furthermore, mutations in the RNA helicase IGHMBP2 which result in a loss of function are attributed to spinal muscular atrophy with respiratory distress type I (SMARD1) (Guenther et al., 2007; Smith et al., 2021) and Charcot-Marie tooth disease type 2 (Cottenie et al., 2014).

Juvenile ALS is an autosomal dominant form of ALS with an age of onset in childhood or adolescence. It is caused by mutations in several genes including alsin rho guanine nucleotide exchange factor (ALS2), senataxin (*SETX*, ALS4), Spastic Paraplegia 11 (*SPG11*, ALS5) and sigma non-opioid intracellular receptor 1 (*SIGMAR1*, ALS16). ALS4, like the adult form of the disease, is characterised by limb weakness, muscle atrophy, and pyramidal features with associated degeneration of motor neurones in the brain and spinal cord (Chen et al., 2004). ALS4 has a long disease duration relative to other forms of ALS. Missense mutations in the C-terminal DEAD-box DNA/RNA helicase domain of *SETX* have been linked to ALS4 through a gain-of-function (Chen et al., 2004). *SETX* missense mutations have also been identified as a causative for neurodegenerative condition ataxia-ocular apraxia 2 (AOA2) through a loss-of-function mechanism (Moreira et al., 2004). So far, over 80 mutations have been identified in *SETX* that have been linked to AOA2, including missense and nonsense loss-of-function mutations within the helicase domain (Richard et al., 2013). SETX interacts with exosome complex component Rrp45, which is linked to RNA quality control, and is dependent on SUMOylation of SETX. This is disrupted in mutations linked with AOA2, but not ALS4 (Richard et al., 2013). SETX has also been linked to other neurodegenerative diseases, including SMA, where SETX overexpression in SMA neuronal models has been shown to rescue a neurodegenerative phenotype through reduction of R-loop formation and DNA damage (Kannan et al., 2018). Cell models harbouring the *C9ORF72* sense repeats also show accumulation of R-loops and double stranded breaks which can also be rescued by SETX overexpression (Walker et al., 2017). R-loop abundance also decreases in ALS4 patient fibroblasts and is seen in immunostaining of patient spinal cord and motor cortex. In ALS4 patient derived cells, R-loops are reduced with associated increases in gene expression and decreased promoter methylation (Richard et al., 2021). ALS4 patients have fewer R-loops in the promoter of BAMBI, a negatively regulator of TGFβ, which is associated with motor neuron dysfunction and disease. Methylation of BAMBI alters the TGFβ pathway, excessive activation of which is linked with degeneration of motor neurones. This provides a link between helicase activity of SETX and impact of a mutation on R-loop levels, with transcriptional dysregulation, and alteration of major signalling pathways linked to motor neuron degeneration (Grunseich et al., 2018).

RNA helicases have also been linked to PD, with DDX1 expression downregulated in specifically in dopaminergic neurons of patients with the G2019S mutation in *LRRK2* (Pallos et al., 2021). Furthermore, DDX10 has been shown to modulate α-synuclein toxicity in PD by sequestering and stabilising its oligomeric forms, with a depletion of DDX10 rescuing associated toxicities (Popova et al., 2021). The RNA helicases DHX30 and DHX37 have also been associated with AD, being significantly downregulated in brain tissue infected with *Porphyromonas* gingivalis, a bacterium associated with AD (Patel et al., 2021). Indeed, a rare frameshift mutation identified in DHX37 has also been linked to AD and novel variants of DHX37 have been linked to structural brain malformations including cortical dysplasia (Karaca et al., 2015). Additionally, DDX49 has been linked to the AD pathogenesis, becoming upregulated during the early stages of disease (Aladeokin et al., 2019).

**FIGURE 3:**
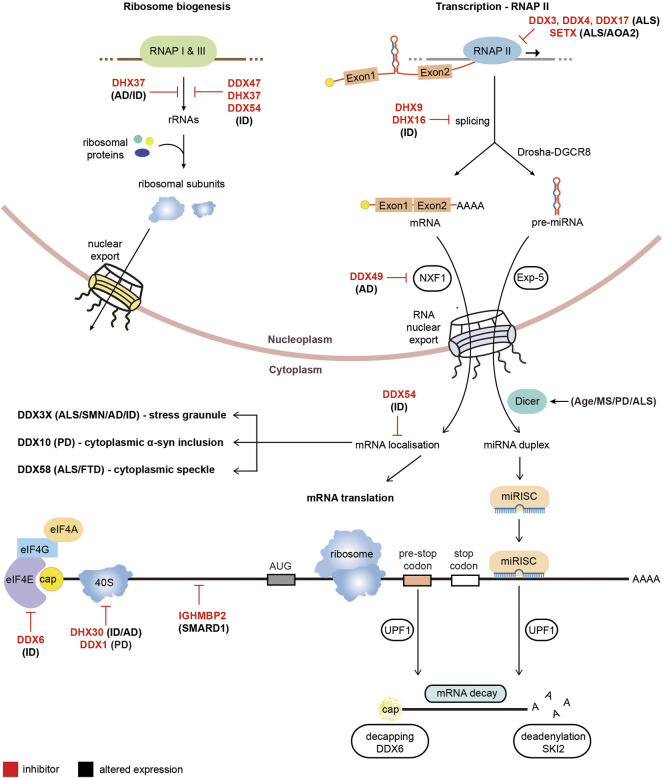
Pathological roles of RNA helicases in non-microsatellite repeat-mediated neurodegeneration. Diagram of RNA metabolic functions with RNA helicases implicated in non-repeat neurodegenerative diseases. RNA helicases labeled in red are prevented from performing their cellular functions while RNA helicases with black text have altered expression in neurodegenerative diseases.

Several RNA helicases are heavily implicated in Intellectual disability (ID) forms of neurodegeneration, including DDX6, DHX30 and IFIH1. DDX6 is an RNA helicase which has functions in enhancing decapping and repressing translation (reviewed in (Ostareck et al., 2014)). As discussed above, it has been implicated in modulating DM1 pathology, however rare *de novo* missense mutations have also been identified as causing ID (Balak et al., 2019). All mutations were found to occur in the short exon encoding to conserved motifs of the RecA domain and appear to affect the role of DDX6 in processing bodies formation and translational repression (Balak et al., 2019). DHX30 is an RNA helicase which so far has not been heavily researched but appears to function in several RNA metabolic processes including mitochondrial ribosome assembly (Antonicka and Shoubridge 2015). *De novo* missense mutations in DHX30 have been implicated in ID and neurodevelopmental disorders (Lessel et al., 2017; Mannucci et al., 2021). The missense mutations linked to ID centre in the conserved amino acids of the helicase core motifs and reduce the helicase activity of DHX30 (Lessel et al., 2017). DHX30 has also been found to bind to the CCG repeats of *FMR1* and depleting DHX30 levels in a *Drosophila* model reduced CGG repeat toxicity, potentially implicating it in FXTAS microsatellite disorder however further studies are required to confirm this association (Malik et al., 2021). Additionally, autoinflammatory responses in the Aicardi-Guotieres or Singleton Merten syndromes and ID have been attributed to mutations which cluster around the ATP binding region of the RNA helicase IFIH1 (Crow et al., 2015; Rice et al., 2020). Finally, exome sequencing has linked several other RNA helicases to rare cases of ID, including DDX47, DDX54, DHX8, DHX16, DHX34, DHX37 and DHX58 (Paine et al., 2019; Järvelä et al., 2021). A diagram summarising the altered functions of RNA helicases in non-repeat neurodegenerative disorders is presented in Figure 3.

## Concluding Remarks

A large number of RNA helicases plays key functions in driving and regulating multiple steps of the mammalian expression of genes. Their roles in unwinding RNA structures is critical in both physiological and pathophysiological conditions. Expansions of short microsatellite sequences of usually 3 to 6 nucleotides are the hallmark of repeat expansions disorders, which mainly encompass neurodegenerative conditions in over 50 diseases (Castelli et al., 2021; Depienne and Mandel 2021). Repeat transcripts are prone to form secondary structures such as G-quadruplexes for GGGGCC and CGG expansions, double-stranded A-form-like helical conformations for CCCCGG expanded hexanucleotides and hairpins for CAG or CTG repeat sequences. They constitute therefore prime substrates for RNA helicases which are required for RAN translation or for generating Dicer-cleaved dsRNA hairpins which mimics siRNAs that further lead to the silencing of the precursor transcripts (Krol et al., 2007; Llamusí and Artero 2008). Multiple studies identified the requirement of the general translation initiation factors eIF4A, eIF4B and eIF4H in the RAN translation of CGG- and GGGGCC-repeat transcripts in FXTAS and C9ORF72-ALS/FTD respectively (Kearse et al., 2016; Green et al., 2017; Tabet et al., 2018; Goodman et al., 2019; Linsalata et al., 2019). The eIF4A protein is essential to the general cellular translation including its prevalence in the preferential translation of highly structured mRNAs encoding oncogenic factors (Wolfe et al., 2014). It has therefore been targeted in multiple drug screenings and developments for cancer applications aiming at killing tumor cells. However none of these compounds have yet reached clinical trial stages likely due to high cytotoxic off-target effects of inhibiting the activity of eIF4A in healthy neighbouring cells. Of particular interest, the partial depletion of *Drosophila* eIF4B and eIF4H1, two translation initiation co-factors stimulating the activity of eIF4A, lead to varying degrees of suppression of the rough eye phenotype in *in vivo* in both FXTAS and C9ORF72-ALS/FTD flies (Goodman et al., 2019; Linsalata et al., 2019) while depletion of the RNA helicase DDX3X leads to a strong inhibition of the neurodegenerative phenotype in FXTAS (Linsalata et al., 2019). Whilst DDX3X plays essential roles in embryogenesis and in immunity against pathogens in adult mice, the reduction of the non-essential eIF4B or eIF4H proteins may provide valid therapeutic strategies. However, safety and efficacy of these interventions have so far only been reported *in vivo* in *Drosophila*. Further studies in mouse models of FXTAS and C9ORF72-ALS/FTD will be necessary prior to validating or infirming these eIF4A co-factors as potential therapeutic targets. The eIF4H gene is deleted in the Williams syndrome, a multisystem developmental disorder, and the specificity and level of depletion in therapeutic approaches is also likely to be critical. On the other hand, improving the UPF1 function through overexpression is generating a large interest in cell and *Drosophila* models of C9RF72-ALS/FTD (Xu et al., 2019; Ortega et al., 2020; Zaepfel et al., 2021) as well as in TDP-43/FUS-ALS (Barmada et al., 2015). However, as previously discussed, mouse studies will now be required for evaluating the therapeutic potential of overexpressing UPF1. It will also be necessary to gain a better understanding of the molecular mechanisms involved in the neuroprotective effects conferred by the up-regulation of UPF1, i.e., NMD-mediated degradation of abnormal diseased transcripts versus other biological function(s) independent of mRNA decay. Due to their potency in modulating the pathophysiological expression of genes, it is anticipated that many more RNA helicases will be targeted in therapeutic approaches for neurodegenerative diseases. However, the specificity of interventions and the control of the depletion or overexpression levels of the manipulated RNA helicases will likely constitute key challenges to overcome before treatments are developed for patients.
