# Supplementary material for: RNA Helicases in Microsatellite Repeat Expansion Disorders and Neurodegeneration
Source: Front Genet. 2022 May 12;13:886563. doi: 10.3389/fgene.2022.886563 (PMC9133428; doi:10.3389/fgene.2022.886563)
Supplement: Supplementary file 1 [file Table1.DOCX]

Supplementary Material

**Supplementary Table 1.** The roles of RNA helicases in gene expression and regulation.

| **RNA Helicase** | **Family** | **Alias** | **Transcription** | **Splicing** | **RNA export** | **Translation** | **RNA decay** | **RNA regulation** | **Viral sensing** | **RNA storage /transport** | **rRNA processing** | **mtRNA** |
| --- | --- | --- | --- | --- | --- | --- | --- | --- | --- | --- | --- | --- |
| DDX1 | SF2 / DEAD | DBP-RB | 1 | 2 | 3, 4 | 5 |  | 6,7 | 8,9 | 10, 11 |  |  |
| DDX2A | SF2 / DEAD | eIF4A1 |  |  |  | 12,13 |  | 14 |  | 16,17 |  |  |
| DDX2B | SF2 / DEAD | eIF4A2 |  |  |  | 12,13 |  | 14,15 |  | 16,17 |  |  |
| DDX3X | SF2 / DEAD | Belle, HLP2 |  | 18 | 19,20 | 20,21 |  | 22,23 | 8,24 | 16,17 |  |  |
| DDX3Y | SF2 / DEAD | DBY |  | 25 |  | 26 |  |  |  |  |  |  |
| DDX4 | SF2 / DEAD | VASA |  |  |  | 27,28 |  |  |  |  |  |  |
| DDX5 | SF2 / DEAD | P68 | 29,30 | 31,32 | 33,34 | 35 | 36,37 | 38,39 | 40 | 41,42 |  |  |
| DDX6 | SF2 / DEAD | RCK |  |  | 43 | 44 | 45,46 | 47,48 |  | 49,50 |  |  |
| DDX10 | SF2 / DEAD | HRH-J8 |  |  |  |  |  |  |  |  | 51,52 |  |
| DDX17 | SF2 / DEAD | P72 | 30,53 | 31,32 | 33,34 |  | 36,37 | 38,39 |  | 41,42 |  |  |
| **RNA Helicase** | **Family** | **Alias** | **Transcription** | **Splicing** | **RNA export** | **Translation** | **RNA decay** | **RNA regulation** | **Viral sensing** | **RNA storage /transport** | **rRNA processing** | **mtRNA** |
| DDX18 | SF2 / DEAD | MrDb |  |  |  |  |  |  |  |  | 54,55 |  |
| DDX19A | SF2 / DEAD | DDX19L |  |  |  |  |  |  | 56 |  |  |  |
| DDX19B | SF2 / DEAD | DBP5 |  |  | 57,58 | 59,60 |  |  |  | 59,61 |  |  |
| DDX20 | SF2 / DEAD | GEMIN3 |  |  |  |  |  | 62,63 |  | 64 |  |  |
| DDX21 | SF2 / DEAD | RH-II/GU | 65 |  | 3 |  |  |  | 8,9 | 65,66 |  |  |
| DDX23 | SF2 / DEAD | SNRNP100 |  | 67,68 |  |  |  | 69 | 70 |  |  |  |
| DDX24 | SF2 / DEAD | --- |  |  |  |  |  |  |  |  | 71 |  |
| DDX25 | SF2 / DEAD | GRTH |  |  | 72,73 | 72,74 |  | 75 |  | 72,74 |  |  |
| DDX27 | SF2 / DEAD | DRS1 |  |  |  |  |  |  |  |  | 76,77 |  |
| DDX28 | SF2 / DEAD | MDDX28 |  |  |  |  |  |  |  |  |  | 78 |
| DDX31 | SF2 / DEAD | PPP1R25 |  |  |  |  |  |  |  |  | 79,80 |  |
| DDX39A | SF2 / DEAD | BAT1L |  | 81 | 82,83 |  |  |  |  |  |  |  |
| DDX39B | SF2 / DEAD | UAP56 |  | 67,68 | 84,85 | 86 |  |  |  | 87 | 88 |  |
| DDX41 | SF2 / DEAD | ABS |  | 88,89 |  |  |  |  | 8,90 | 91 |  |  |
| **RNA Helicase** | **Family** | **Alias** | **Transcription** | **Splicing** | **RNA export** | **Translation** | **RNA decay** | **RNA regulation** | **Viral sensing** | **RNA storage /transport** | **rRNA processing** | **mtRNA** |
| DDX42 | SF2 / DEAD | RHELP |  | 88 |  |  |  |  |  |  |  |  |
| DDX43 | SF2 / DEAD | HAGE |  |  |  | 92 |  |  |  |  |  |  |
| DDX46 | SF2 / DEAD | PRPF5 |  | 67,68 |  |  |  |  |  |  |  |  |
| DDX47 | SF2 / DEAD | RRP3 |  |  |  |  |  |  |  |  | 93,94 |  |
| DDX48 | SF2 / DEAD | eIF4A3 | 95,96 | 96 | 97,98 |  |  | 98,99 |  | 100 |  |  |
| DDX49 | SF2 / DEAD | DBP8 |  |  | 101 | 101 |  |  |  | 102,103 | 101 |  |
| DDX50 | SF2 / DEAD | RH-II/GuB |  |  |  |  |  |  |  |  | 104 |  |
| DDX51 | SF2 / DEAD | --- |  |  |  |  |  |  |  | 105,106 |  |  |
| DDX52 | SF2 / DEAD | ROK1 |  |  |  |  |  |  |  | 107,108 |  |  |
| DDX53 | SF2 / DEAD | CAGE |  |  |  |  |  |  |  |  |  |  |
| DDX54 | SF2 / DEAD | DP97 |  |  |  |  |  |  |  | 109 |  |  |
| DDX55 | SF2 / DEAD | KIAAI595 |  |  |  |  |  |  |  | 110,111 |  |  |
| DDX56 | SF2 / DEAD | NOH61 |  |  |  |  |  |  | 112 | 113 |  |  |
| DDX59 | SF2 / DEAD | ZNHIT5 |  |  |  |  |  |  |  |  |  |  |
| DHX8 | SF2 / DExH | PRPF22 |  | 67,68 |  |  |  |  |  |  |  |  |
| **Gene** | **Superfamily / Family** | **Alias** | **Transcription** | **Splicing** | **RNA export** | **Translation** | **RNA decay** | **RNA regulation** | **Viral sensing** | **Storage / Transport** | **rRNA processing** | **mtRNA** |
| DHX9 | SF2 / DExH | RHA | 53,114 | 88,115 | 116,117 | 118,119 |  | 120,121 | 8,122 |  |  |  |
| DHX15 | SF2 / DExH | DBP1 |  | 67,68 |  | 123 |  |  | 70,124 |  |  |  |
| DHX16 | SF2 / DExH | DBP2 |  | 67,68 |  |  |  |  |  |  |  |  |
| DHX29 | SF2 / DExH | --- |  |  |  | 125,126 |  |  | 127 |  |  |  |
| DHX30 | SF2 / DExH | KIAA0890 |  |  |  | 128,129 |  |  |  |  |  | 128,130 |
| DHX32 | SF2 / DExH | DHLP1 |  |  |  |  |  |  |  |  | 131,132 | 131,132 |
| DHX33 | SF2 / DExH | --- |  |  |  | 133 |  |  | 134,135 |  |  |  |
| DHX34 | SF2 / DExH | KIAA0134 |  |  |  |  | 136, 137 |  |  |  |  |  |
| DHX35 | SF2 / DExH | KAIK0875 |  | 88 |  |  | 138 |  |  |  |  |  |
| DHX36 | SF2 / DExH | RHAU |  |  |  | 139,140 |  | 139,140 | 8,9 | 141,142 |  |  |
| DHX37 | SF2 / DExH | KIAA1517 |  |  |  |  |  |  |  |  | 143 |  |
| DHX38 | SF2 / DExH | PRP16 |  | 67,68 |  |  |  |  |  |  |  |  |
| DHX40 | SF2 / DExH | ARG147 |  |  |  |  |  |  |  |  |  |  |
| DHX57 | SF2 / DExH | --- |  |  |  |  |  |  |  |  |  |  |
| **Gene** | **Superfamily / Family** | **Alias** | **Transcription** | **Splicing** | **RNA export** | **Translation** | **RNA decay** | **RNA regulation** | **Viral sensing** | **Storage / Transport** | **rRNA processing** | **mtRNA** |
| SKIV2L | SF2 / DExH | DDX13 |  |  |  | 144,145 |  |  |  |  |  |  |
| MTREX | SF2 / DExH | SKIV2L2 |  |  |  |  | 144 |  |  |  | 146,147 |  |
| SNRNP200 | SF2 / DExH | HELIC2 |  | 67,68 |  |  |  |  | 147 |  |  |  |
| DDX60 | SF2 / DExH | FLJ20035 |  |  |  |  |  |  | 148,149 |  |  |  |
| SUPV3L1 | SF2 / DExH | SUV3 |  |  |  |  |  |  | 150 |  |  | 150,151 |
| DICER1 | SF2 / DExH | HERNA |  |  |  |  | 152, 153 |  | 254 |  |  |  |
| DDX58 | SF2 / DExH | RIG-1 |  |  |  |  |  |  | 155,156 |  |  |  |
| DHX58 | SF2 / DExH | LGP2 |  |  |  |  |  |  | 155,156 |  |  |  |
| IFIH1 | SF2 / DExH | MDA-5 |  |  |  |  |  |  | 155,156 |  |  |  |
| UPF1 | SF1 / Upf1-like | NORF1 |  |  |  |  | 157, 158 |  |  |  |  |  |
| MOV10 | SF1 / Upf1-like | FSAP113 |  |  | 159 | 160,161 | 162 | 163,164 | 165 | 164 |  |  |
| MOV10L1 | SF1 / Upf1-like | CHAMP |  |  |  |  |  | 166,167 |  |  |  |  |
| AQR | SF1 / Upf1-like | IBP160 |  | 168 |  |  |  |  |  |  |  |  |
| **Gene** | **Superfamily / Family** | **Alias** | **Transcription** | **Splicing** | **RNA export** | **Translation** | **RNA decay** | **RNA regulation** | **Viral sensing** | **Storage / Transport** | **rRNA processing** | **mtRNA** |
| IGHMBP2 | SF1 / Upf1-like | DMUBP2 |  |  |  | 169 |  |  |  |  |  |  |
| SETX | SF1 / Upf1-like | AQA2 | 170 |  |  |  |  |  | 171 |  |  |  |

**Supplementary References**

1. Ishaq M, Ma L, Wu X, Mu Y, Pan J, Hu J, et al. The DEAD-box RNA helicase DDX1 interacts with RelA and enhances nuclear factor kappaB-mediated transcription. J Cell Biochem. 2009;106(2):296-305.

2. Germain DR, Li L, Hildebrandt MR, Simmonds AJ, Hughes SC, Godbout R. Loss of the Drosophila melanogaster DEAD box protein Ddx1 leads to reduced size and aberrant gametogenesis. Dev Biol. 2015;407(2):232-45.

3. Yasuda-Inoue M, Kuroki M, Ariumi Y. Distinct DDX DEAD-box RNA helicases cooperate to modulate the HIV-1 Rev function. Biochem Biophys Res Commun. 2013;434(4):803-8.

4. Perez-Gonzalez A, Pazo A, Navajas R, Ciordia S, Rodriguez-Frandsen A, Nieto A. hCLE/C14orf166 associates with DDX1-HSPC117-FAM98B in a novel transcription-dependent shuttling RNA-transporting complex. PLoS One. 2014;9(3):e90957.

5. Pazo A, Perez-Gonzalez A, Oliveros JC, Huarte M, Chavez JP, Nieto A. hCLE/RTRAF-HSPC117-DDX1-FAM98B: A New Cap-Binding Complex That Activates mRNA Translation. Front Physiol. 2019;10:92.

6. Han C, Liu Y, Wan G, Choi HJ, Zhao L, Ivan C, et al. The RNA-binding protein DDX1 promotes primary microRNA maturation and inhibits ovarian tumor progression. Cell Rep. 2014;8(5):1447-60.

7. Gregory RI, Yan KP, Amuthan G, Chendrimada T, Doratotaj B, Cooch N, et al. The Microprocessor complex mediates the genesis of microRNAs. Nature. 2004;432(7014):235-40.

8. Mojzesz M, Klak K, Wojtal P, Adamek M, Podlasz P, Chmielewska-Krzesinska M, et al. Viral infection-induced changes in the expression profile of non-RLR DExD/H-box RNA helicases (DDX1, DDX3, DHX9, DDX21 and DHX36) in zebrafish and common carp. Fish Shellfish Immunol. 2020;104:62-73.

9. Zhang Z, Kim T, Bao M, Facchinetti V, Jung SY, Ghaffari AA, et al. DDX1, DDX21, and DHX36 helicases form a complex with the adaptor molecule TRIF to sense dsRNA in dendritic cells. Immunity. 2011;34(6):866-78.

10. Vessey JP, Amadei G, Burns SE, Kiebler MA, Kaplan DR, Miller FD. An asymmetrically localized Staufen2-dependent RNA complex regulates maintenance of mammalian neural stem cells. Cell Stem Cell. 2012;11(4):517-28.

11. Kunde SA, Musante L, Grimme A, Fischer U, Muller E, Wanker EE, et al. The X-chromosome-linked intellectual disability protein PQBP1 is a component of neuronal RNA granules and regulates the appearance of stress granules. Hum Mol Genet. 2011;20(24):4916-31.

12. Lu WT, Wilczynska A, Smith E, Bushell M. The diverse roles of the eIF4A family: you are the company you keep. Biochem Soc Trans. 2014;42(1):166-72.

13. Pestova TV, Kolupaeva VG. The roles of individual eukaryotic translation initiation factors in ribosomal scanning and initiation codon selection. Genes Dev. 2 002;16(22):2906-22.

14. Jonas S, Izaurralde E. Towards a molecular understanding of microRNA-mediated gene silencing. Nat Rev Genet. 2015;16(7):421-33.

15. Meijer HA, Kong YW, Lu WT, Wilczynska A, Spriggs RV, Robinson SW, et al. Translational repression and eIF4A2 activity are critical for microRNA- mediated gene regulation. Science. 2013;340(6128):82-5.

16. Hooper C, Hilliker A. Packing them up and dusting them off: RNA helicases and mRNA storage. Biochim Biophys Acta. 2013;1829(8):824-34.

17. Hilliker A. Analysis of RNA helicases in P-bodies and stress granules. Methods Enzymol. 2012;511:323-46.

18. Burckin T, Nagel R, Mandel-Gutfreund Y, Shiue L, Clark TA, Chong JL, et al. Exploring functional relationships between components of the gene expression machinery. Nat Struct Mol Biol. 2005;12(2):175-82.

19. Choi YJ, Lee SG. The DEAD-box RNA helicase DDX3 interacts with DDX5, co-localizes with it in the cytoplasm during the G2/M phase of the cycle, and affects its shuttling during mRNP export. J Cell Biochem. 2012;113(3):985-96.

20. Lai MC, Lee YH, Tarn WY. The DEAD-box RNA helicase DDX3 associates with export messenger ribonucleoproteins as well as tip-associated protein and participates in translational control. Mol Biol Cell. 2008;19(9):3847-58.

21. Soto-Rifo R, Rubilar PS, Limousin T, de Breyne S, Decimo D, Ohlmann T. DEAD-box protein DDX3 associates with eIF4F to promote translation of selected mRNAs. EMBO J. 2012;31(18):3745-56.

22. Krol J, Krol I, Alvarez CP, Fiscella M, Hierlemann A, Roska B, et al. A network comprising short and long noncoding RNAs and RNA helicase controls mouse retina architecture. Nat Commun. 2015;6:7305.

23. Kasim V, Wu S, Taira K, Miyagishi M. Determination of the role of DDX3 a factor involved in mammalian RNAi pathway using an shRNA-expression library. PLoS One. 2013;8(3):e59445.

24. Lai MC, Sun HS, Wang SW, Tarn WY. DDX3 functions in antiviral innate immunity through translational control of PACT. FEBS J. 2016;283(1):88-101.

25. Vakilian H, Mirzaei M, Sharifi Tabar M, Pooyan P, Habibi Rezaee L, Parker L, et al. DDX3Y, a Male-Specific Region of Y Chromosome Gene, May Modulate Neuronal Differentiation. J Proteome Res. 2015;14(9):3474-83.

26. Venkataramanan S, Gadek M, Calviello L, Wilkins K, Floor SN. DDX3X and DDX3Y are redundant in protein synthesis. RNA. 2021;27(12):1577-88.

27. Markussen FH, Breitwieser W, Ephrussi A. Efficient translation and phosphorylation of Oskar require Oskar protein and the RNA helicase Vasa. Cold Spring Harb Symp Quant Biol. 1997;62:13-7.

28. Yajima M, Wessel GM. Essential elements for translation: the germline factor Vasa functions broadly in somatic cells. Development. 2015;142(11):1960-70.

29. Zonta E, Bittencourt D, Samaan S, Germann S, Dutertre M, Auboeuf D. The RNA helicase DDX5/p68 is a key factor promoting c-fos expression at different levels from transcription to mRNA export. Nucleic Acids Res. 2013;41(1):554-64.

30. Germann S, Gratadou L, Zonta E, Dardenne E, Gaudineau B, Fougere M, et al. Dual role of the ddx5/ddx17 RNA helicases in the control of the pro-migratory NFAT5 transcription factor. Oncogene. 2012;31(42):4536-49.

31. Dardenne E, Polay Espinoza M, Fattet L, Germann S, Lambert MP, Neil H, et al. RNA helicases DDX5 and DDX17 dynamically orchestrate transcription, miRNA, and splicing programs in cell differentiation. Cell Rep. 2014;7(6):1900-13.

32. Camats M, Guil S, Kokolo M, Bach-Elias M. P68 RNA helicase (DDX5) alters activity of cis- and trans-acting factors of the alternative splicing of H-Ras. PLoS One. 2008;3(8):e2926.

33. Ma WK, Tran EJ. Measuring helicase inhibition of the DEAD-box protein Dbp2 by Yra1. Methods Mol Biol. 2015;1259:183-97.

34. Wang H, Gao X, Huang Y, Yang J, Liu ZR. P68 RNA helicase is a nucleocytoplasmic shuttling protein. Cell Res. 2009;19(12):1388-400.

35. Sun J, Wu G, Pastor F, Rahman N, Wang WH, Zhang Z, et al. RNA helicase DDX5 enables STAT1 mRNA translation and interferon signalling in hepatitis B virus replicating hepatocytes. Gut. 2021.

36. Geissler V, Altmeyer S, Stein B, Uhlmann-Schiffler H, Stahl H. The RNA helicase Ddx5/p68 binds to hUpf3 and enhances NMD of Ddx17/p72 and Smg5 mRNA. Nucleic Acids Res. 2013;41(16):7875-88.

37. Bond AT, Mangus DA, He F, Jacobson A. Absence of Dbp2p alters both nonsense-mediated mRNA decay and rRNA processing. Mol Cell Biol. 2001;21(21):7366-79.

38. Wang D, Huang J, Hu Z. RNA helicase DDX5 regulates microRNA expression and contributes to cytoskeletal reorganization in basal breast cancer cells. Mol Cell Proteomics. 2012;11(2):M111 011932.

39. Salzman DW, Shubert-Coleman J, Furneaux H. P68 RNA helicase unwinds the human let-7 microRNA precursor duplex and is required for let-7-directed silencing of gene expression. J Biol Chem. 2007;282(45):32773-9.

40. Dixon CR, Malik P, de Las Heras JI, Saiz-Ros N, de Lima Alves F, Tingey M, et al. STING nuclear partners contribute to innate immune signaling responses. iScience. 2021;24(9):103055.

41. Jalal C, Uhlmann-Schiffler H, Stahl H. Redundant role of DEAD box proteins p68 (Ddx5) and p72/p82 (Ddx17) in ribosome biogenesis and cell proliferation. Nucleic Acids Res. 2007;35(11):3590-601.

42. Fuller-Pace FV. The DEAD box proteins DDX5 (p68) and DDX17 (p72): multi-tasking transcriptional regulators. Biochim Biophys Acta. 2013;1829(8):756- 63.

43. Smillie DA, Sommerville J. RNA helicase p54 (DDX6) is a shuttling protein involved in nuclear assembly of stored mRNP particles. J Cell Sci. 2002;115(Pt 2):395-407.

44. Wang Y, Arribas-Layton M, Chen Y, Lykke-Andersen J, Sen GL. DDX6 Orchestrates Mammalian Progenitor Function through the mRNA Degradation and Translation Pathways. Mol Cell. 2015;60(1):118-30.

45. Hu G, McQuiston T, Bernard A, Park YD, Qiu J, Vural A, et al. A conserved mechanism of TOR-dependent RCK-mediated mRNA degradation regulates autophagy. Nat Cell Biol. 2015;17(7):930-42.

46. Coller JM, Tucker M, Sheth U, Valencia-Sanchez MA, Parker R. The DEAD box helicase, Dhh1p, functions in mRNA decapping and interacts with both the decapping and deadenylase complexes. RNA. 2001;7(12):1717-27.

47. Rouya C, Siddiqui N, Morita M, Duchaine TF, Fabian MR, Sonenberg N. Human DDX6 effects miRNA-mediated gene silencing via direct binding to CNOT1. RNA. 2014;20(9):1398-409.

48. Chen Y, Boland A, Kuzuoglu-Ozturk D, Bawankar P, Loh B, Chang CT, et al. A DDX6-CNOT1 complex and W-binding pockets in CNOT9 reveal direct links between miRNA target recognition and silencing. Mol Cell. 2014;54(5):737-50.

49. Ayache J, Benard M, Ernoult-Lange M, Minshall N, Standart N, Kress M, et al. P-body assembly requires DDX6 repression complexes rather than decay or Ataxin2/2L complexes. Mol Biol Cell. 2015;26(14):2579-95.

50. Nicklas S, Okawa S, Hillje AL, Gonzalez-Cano L, Del Sol A, Schwamborn JC. The RNA helicase DDX6 regulates cell-fate specification in neural stem cells via miRNAs. Nucleic Acids Res. 2015;43(5):2638-54.

51. Soltanieh S, Osheim YN, Spasov K, Trahan C, Beyer AL, Dragon F. DEAD-box RNA helicase Dbp4 is required for small-subunit processome formation and function. Mol Cell Biol. 2015;35(5):816-30.

52. Soltanieh S, Lapensee M, Dragon F. Nucleolar proteins Bfr2 and Enp2 interact with DEAD-box RNA helicase Dbp4 in two different complexes. Nucleic Acids Res. 2014;42(5):3194-206.

53. Fuller-Pace FV. DExD/H box RNA helicases: multifunctional proteins with important roles in transcriptional regulation. Nucleic Acids Res. 2006;34(15):4206- 15.

54. Emery B, de la Cruz J, Rocak S, Deloche O, Linder P. Has1p, a member of the DEAD-box family, is required for 40S ribosomal subunit biogenesis in Saccharomyces cerevisiae. Mol Microbiol. 2004;52(1):141-58.

55. Dembowski JA, Kuo B, Woolford JL, Jr. Has1 regulates consecutive maturation and processing steps for assembly of 60S ribosomal subunits. Nucleic Acids Res. 2013;41(16):7889-904.

56. Li J, Hu L, Liu Y, Huang L, Mu Y, Cai X, et al. DDX19A Senses Viral RNA and Mediates NLRP3-Dependent Inflammasome Activation. J Immunol. 2015;195(12):5732-49.

57. Valkov E, Dean JC, Jani D, Kuhlmann SI, Stewart M. Structural basis for the assembly and disassembly of mRNA nuclear export complexes. Biochim Biophys Acta. 2012;1819(6):578-92.

58. Ledoux S, Guthrie C. Regulation of the Dbp5 ATPase cycle in mRNP remodeling at the nuclear pore: a lively new paradigm for DEAD-box proteins. Genes Dev. 2011;25(11):1109-14.

59. Tieg B, Krebber H. Dbp5 - from nuclear export to translation. Biochim Biophys Acta. 2013;1829(8):791-8.

60. Gross T, Siepmann A, Sturm D, Windgassen M, Scarcelli JJ, Seedorf M, et al. The DEAD-box RNA helicase Dbp5 functions in translation termination. Science. 2007;315(5812):646-9.

61. Scarcelli JJ, Viggiano S, Hodge CA, Heath CV, Amberg DC, Cole CN. Synthetic genetic array analysis in Saccharomyces cerevisiae provides evidence for an interaction between RAT8/DBP5 and genes encoding P-body components. Genetics. 2008;179(4):1945-55.

62. Takata A, Otsuka M, Yoshikawa T, Kishikawa T, Kudo Y, Goto T, et al. A miRNA machinery component DDX20 controls NF-kappaB via microRNA-140 function. Biochem Biophys Res Commun. 2012;420(3):564-9.

63. Hutvagner G, Zamore PD. A microRNA in a multiple-turnover RNAi enzyme complex. Science. 2002;297(5589):2056-60.

64. Todd AG, Morse R, Shaw DJ, McGinley S, Stebbings H, Young PJ. SMN, Gemin2 and Gemin3 associate with beta-actin mRNA in the cytoplasm of neuronal cells in vitro. J Mol Biol. 2010;401(5):681-9.

65. Calo E, Flynn RA, Martin L, Spitale RC, Chang HY, Wysocka J. RNA helicase DDX21 coordinates transcription and ribosomal RNA processing. Nature. 2015;518(7538):249-53.

66. Zhang Y, Baysac KC, Yee LF, Saporita AJ, Weber JD. Elevated DDX21 regulates c-Jun activity and rRNA processing in human breast cancers. Breast Cancer Res. 2014;16(5):449.

67. Koodathingal P, Staley JP. Splicing fidelity: DEAD/H-box ATPases as molecular clocks. RNA Biol. 2013;10(7):1073-9.

68. Chang TH, Tung L, Yeh FL, Chen JH, Chang SL. Functions of the DExD/H-box proteins in nuclear pre-mRNA splicing. Biochim Biophys Acta. 2013;1829(8):764-74.

69. Yin J, Park G, Lee JE, Choi EY, Park JY, Kim TH, et al. DEAD-box RNA helicase DDX23 modulates glioma malignancy via elevating miR-21 biogenesis. Brain. 2015;138(Pt 9):2553-70.

70. Ruan J, Cao Y, Ling T, Li P, Wu S, Peng D, et al. DDX23, an Evolutionary Conserved dsRNA Sensor, Participates in Innate Antiviral Responses by Pairing With TRIF or MAVS. Front Immunol. 2019;10:2202.

71. Pratte D, Singh U, Murat G, Kressler D. Mak5 and Ebp2 act together on early pre-60S particles and their reduced functionality bypasses the requirement for the essential pre-60S factor Nsa1. PLoS One. 2013;8(12):e82741.

72. Sheng Y, Tsai-Morris CH, Gutti R, Maeda Y, Dufau ML. Gonadotropin-regulated testicular RNA helicase (GRTH/Ddx25) is a transport protein involved in gene-specific mRNA export and protein translation during spermatogenesis. J Biol Chem. 2006;281(46):35048-56.

73. Tsai-Morris CH, Sheng Y, Lee E, Lei KJ, Dufau ML. Gonadotropin-regulated testicular RNA helicase (GRTH/Ddx25) is essential for spermatid development and completion of spermatogenesis. Proc Natl Acad Sci U S A. 2004;101(17):6373-8.

74. Tsai-Morris CH, Sato H, Gutti R, Dufau ML. Role of gonadotropin regulated testicular RNA helicase (GRTH/Ddx25) on polysomal associated mRNAs in mouse testis. PLoS One. 2012;7(3):e32470.

75. Dai L, Tsai-Morris CH, Sato H, Villar J, Kang JH, Zhang J, et al. Testis-specific miRNA-469 up-regulated in gonadotropin-regulated testicular RNA helicase (GRTH/DDX25)-null mice silences transition protein 2 and protamine 2 messages at sites within coding region: implications of its role in germ cell development. J Biol Chem. 2011;286(52):44306-18.

76. Kellner M, Rohrmoser M, Forne I, Voss K, Burger K, Muhl B, et al. DEAD-box helicase DDX27 regulates 3' end formation of ribosomal 47S RNA and stably associates with the PeBoW-complex. Exp Cell Res. 2015;334(1):146-59.

77. Ripmaster TL, Vaughn GP, Woolford JL, Jr. A putative ATP-dependent RNA helicase involved in Saccharomyces cerevisiae ribosome assembly. Proc Natl Acad Sci U S A. 1992;89(23):11131-5.

78. Tu YT, Barrientos A. The Human Mitochondrial DEAD-Box Protein DDX28 Resides in RNA Granules and Functions in Mitoribosome Assembly. Cell Rep. 2015;10(6):854-64.

79. Daugeron MC, Linder P. Dbp7p, a putative ATP-dependent RNA helicase from Saccharomyces cerevisiae, is required for 60S ribosomal subunit assembly. RNA. 1998;4(5):566-81.

80. Fukawa T, Ono M, Matsuo T, Uehara H, Miki T, Nakamura Y, et al. DDX31 regulates the p53-HDM2 pathway and rRNA gene transcription through its interaction with NPM1 in renal cell carcinomas. Cancer Res. 2012;72(22):5867-77.

81. Strasser K, Masuda S, Mason P, Pfannstiel J, Oppizzi M, Rodriguez-Navarro S, et al. TREX is a conserved complex coupling transcription with messenger RNA export. Nature. 2002;417(6886):304-8.

82. Thomas M, Lischka P, Muller R, Stamminger T. The cellular DExD/H-box RNA-helicases UAP56 and URH49 exhibit a CRM1-independent nucleocytoplasmic shuttling activity. PLoS One. 2011;6(7):e22671.

83. Yamazaki T, Fujiwara N, Yukinaga H, Ebisuya M, Shiki T, Kurihara T, et al. The closely related RNA helicases, UAP56 and URH49, preferentially form distinct mRNA export machineries and coordinately regulate mitotic progression. Mol Biol Cell. 2010;21(16):2953-65.

84. Chang CT, Hautbergue GM, Walsh MJ, Viphakone N, van Dijk TB, Philipsen S, et al. Chtop is a component of the dynamic TREX mRNA export complex. EMBO J. 2013;32(3):473-86.

85. Hautbergue GM, Hung ML, Walsh MJ, Snijders AP, Chang CT, Jones R, et al. UIF, a New mRNA export adaptor that works together with REF/ALY, requires FACT for recruitment to mRNA. Curr Biol. 2009;19(22):1918-24.

86. Awasthi S, Chakrapani B, Mahesh A, Chavali PL, Chavali S, Dhayalan A. DDX39B promotes translation through regulation of pre-ribosomal RNA levels. RNA Biol. 2018;15(9):1157-66.

87. Meignin C, Davis I. UAP56 RNA helicase is required for axis specification and cytoplasmic mRNA localization in Drosophila. Dev Biol. 2008;315(1):89-98.

88. Ilagan JO, Chalkley RJ, Burlingame AL, Jurica MS. Rearrangements within human spliceosomes captured after exon ligation. RNA. 2013;19(3):400-12.

89. Polprasert C, Schulze I, Sekeres MA, Makishima H, Przychodzen B, Hosono N, et al. Inherited and Somatic Defects in DDX41 in Myeloid Neoplasms. Cancer Cell. 2015;27(5):658-70.

90. Moriyama M, Koshiba T, Ichinohe T. Influenza A virus M2 protein triggers mitochondrial DNA-mediated antiviral immune responses. Nat Commun. 2019;10(1):4624.

91. Irion U, Leptin M. Developmental and cell biological functions of the Drosophila DEAD-box protein abstrakt. Curr Biol. 1999;9(23):1373-81.

92. Linley AJ, Mathieu MG, Miles AK, Rees RC, McArdle SE, Regad T. The helicase HAGE expressed by malignant melanoma-initiating cells is required for tumor cell proliferation in vivo. J Biol Chem. 2012;287(17):13633-43.

93. Sekiguchi T, Hayano T, Yanagida M, Takahashi N, Nishimoto T. NOP132 is required for proper nucleolus localization of DEAD-box RNA helicase DDX47. Nucleic Acids Res. 2006;34(16):4593-608.

94. O'Day CL, Chavanikamannil F, Abelson J. 18S rRNA processing requires the RNA helicase-like protein Rrp3. Nucleic Acids Res. 1996;24(16):3201-7.

95. Wang Z, Murigneux V, Le Hir H. Transcriptome-wide modulation of splicing by the exon junction complex. Genome Biol. 2014;15(12):551.

96. Michelle L, Cloutier A, Toutant J, Shkreta L, Thibault P, Durand M, et al. Proteins associated with the exon junction complex also control the alternative splicing of apoptotic regulators. Mol Cell Biol. 2012;32(5):954-67.

97. Choe J, Ryu I, Park OH, Park J, Cho H, Yoo JS, et al. eIF4AIII enhances translation of nuclear cap-binding complex-bound mRNAs by promoting disruption of secondary structures in 5'UTR. Proc Natl Acad Sci U S A. 2014;111(43):E4577-86.

98. Giorgi C, Yeo GW, Stone ME, Katz DB, Burge C, Turrigiano G, et al. The EJC factor eIF4AIII modulates synaptic strength and neuronal protein expression. Cell. 2007;130(1):179-91.

99. Palacios IM, Gatfield D, St Johnston D, Izaurralde E. An eIF4AIII-containing complex required for mRNA localization and nonsense-mediated mRNA decay. Nature. 2004;427(6976):753-7.

100. Kressler D, de la Cruz J, Rojo M, Linder P. Fal1p is an essential DEAD-box protein involved in 40S-ribosomal-subunit biogenesis in Saccharomyces cerevisiae. Mol Cell Biol. 1997;17(12):7283-94.

101. Awasthi S, Verma M, Mahesh A, MI KK, Govindaraju G, Rajavelu A, et al. DDX49 is an RNA helicase that affects translation by regulating mRNA export and the levels of pre-ribosomal RNA. Nucleic Acids Res. 2018;46(12):6304-17.

102. Daugeron MC, Linder P. Characterization and mutational analysis of yeast Dbp8p, a putative RNA helicase involved in ribosome biogenesis. Nucleic Acids Res. 2001;29(5):1144-55.

103. Weaver PL, Sun C, Chang TH. Dbp3p, a putative RNA helicase in Saccharomyces cerevisiae, is required for efficient pre-rRNA processing predominantly at site A3. Mol Cell Biol. 1997;17(3):1354-65.

104. De Silva D, Fontanesi F, Barrientos A. The DEAD box protein Mrh4 functions in the assembly of the mitochondrial large ribosomal subunit. Cell Metab. 2013;18(5):712-25.

105. Srivastava L, Lapik YR, Wang M, Pestov DG. Mammalian DEAD box protein Ddx51 acts in 3' end maturation of 28S rRNA by promoting the release of U8 snoRNA. Mol Cell Biol. 2010;30(12):2947-56.

106. Kressler D, de la Cruz J, Rojo M, Linder P. Dbp6p is an essential putative ATP-dependent RNA helicase required for 60S-ribosomal-subunit assembly in Saccharomyces cerevisiae. Mol Cell Biol. 1998;18(4):1855-65.

107. Martin R, Hackert P, Ruprecht M, Simm S, Bruning L, Mirus O, et al. A pre-ribosomal RNA interaction network involving snoRNAs and the Rok1 helicase. RNA. 2014;20(8):1173-82.

108. Venema J, Bousquet-Antonelli C, Gelugne JP, Caizergues-Ferrer M, Tollervey D. Rok1p is a putative RNA helicase required for rRNA processing. Mol Cell Biol. 1997;17(6):3398-407.

109. Burger F, Daugeron MC, Linder P. Dbp10p, a putative RNA helicase from Saccharomyces cerevisiae, is required for ribosome biogenesis. Nucleic Acids Res. 2000;28(12):2315-23.

110. de la Cruz J, Kressler D, Rojo M, Tollervey D, Linder P. Spb4p, an essential putative RNA helicase, is required for a late step in the assembly of 60S ribosomal subunits in Saccharomyces cerevisiae. RNA. 1998;4(10):1268-81.

111. Sachs AB, Davis RW. Translation initiation and ribosomal biogenesis: involvement of a putative rRNA helicase and RPL46. Science. 1990;247(4946):1077-9.

112. Pirincal A, Turan K. Human DDX56 protein interacts with influenza A virus NS1 protein and stimulates the virus replication. Genet Mol Biol. 2021;44(1):e20200158.

113. Daugeron MC, Kressler D, Linder P. Dbp9p, a putative ATP-dependent RNA helicase involved in 60S-ribosomal-subunit biogenesis, functionally interacts with Dbp6p. RNA. 2001;7(9):1317-34.

114. Aratani S, Fujii R, Oishi T, Fujita H, Amano T, Ohshima T, et al. Dual roles of RNA helicase A in CREB-dependent transcription. Mol Cell Biol. 2001;21(14):4460-9.

115. Reenan RA, Hanrahan CJ, Ganetzky B. The mle(napts) RNA helicase mutation in drosophila results in a splicing catastrophe of the para Na+ channel transcript in a region of RNA editing. Neuron. 2000;25(1):139-49.

116. Reddy TR, Tang H, Xu W, Wong-Staal F. Sam68, RNA helicase A and Tap cooperate in the post-transcriptional regulation of human immunodeficiency virus and type D retroviral mRNA. Oncogene. 2000;19(32):3570-5.

117. Tang H, Wong-Staal F. Specific interaction between RNA helicase A and Tap, two cellular proteins that bind to the constitutive transport element of type D retrovirus. J Biol Chem. 2000;275(42):32694-700.

118. Manojlovic Z, Stefanovic B. A novel role of RNA helicase A in regulation of translation of type I collagen mRNAs. RNA. 2012;18(2):321-34.

119. Hartman TR, Qian S, Bolinger C, Fernandez S, Schoenberg DR, Boris-Lawrie K. RNA helicase A is necessary for translation of selected messenger RNAs. Nat Struct Mol Biol. 2006;13(6):509-16.

120. Kawai S, Amano A. BRCA1 regulates microRNA biogenesis via the DROSHA microprocessor complex. J Cell Biol. 2012;197(2):201-8.

121. Robb GB, Rana TM. RNA helicase A interacts with RISC in human cells and functions in RISC loading. Mol Cell. 2007;26(4):523-37.

122. Ng YC, Chung WC, Kang HR, Cho HJ, Park EB, Kang SJ, et al. A DNA-sensing-independent role of a nuclear RNA helicase, DHX9, in stimulation of NF- kappaB-mediated innate immunity against DNA virus infection. Nucleic Acids Res. 2018;46(17):9011-26.

123. Inesta-Vaquera F, Chaugule VK, Galloway A, Chandler L, Rojas-Fernandez A, Weidlich S, et al. DHX15 regulates CMTR1-dependent gene expression and cell proliferation. Life Sci Alliance. 2018;1(3):e201800092.

124. Xing J, Zhou X, Fang M, Zhang E, Minze LJ, Zhang Z. DHX15 is required to control RNA virus-induced intestinal inflammation. Cell Rep. 2021;35(12):109205.

125. Dhote V, Sweeney TR, Kim N, Hellen CU, Pestova TV. Roles of individual domains in the function of DHX29, an essential factor required for translation of structured mammalian mRNAs. Proc Natl Acad Sci U S A. 2012;109(46):E3150-9.

126. Pisareva VP, Pisarev AV, Komar AA, Hellen CU, Pestova TV. Translation initiation on mammalian mRNAs with structured 5'UTRs requires DExH-box protein DHX29. Cell. 2008;135(7):1237-50.

127. Sugimoto N, Mitoma H, Kim T, Hanabuchi S, Liu YJ. Helicase proteins DHX29 and RIG-I cosense cytosolic nucleic acids in the human airway system. Proc Natl Acad Sci U S A. 2014;111(21):7747-52.

128. Bosco B, Rossi A, Rizzotto D, Hamadou MH, Bisio A, Giorgetta S, et al. DHX30 Coordinates Cytoplasmic Translation and Mitochondrial Function Contributing to Cancer Cell Survival. Cancers (Basel). 2021;13(17).

129. Rizzotto D, Zaccara S, Rossi A, Galbraith MD, Andrysik Z, Pandey A, et al. Nutlin-Induced Apoptosis Is Specified by a Translation Program Regulated by PCBP2 and DHX30. Cell Rep. 2020;30(13):4355-69 e6.

130. Antonicka H, Shoubridge EA. Mitochondrial RNA Granules Are Centers for Posttranscriptional RNA Processing and Ribosome Biogenesis. Cell Rep. 2015;10(6):920-32.

131. Abdelhaleem M. The novel helicase homologue DDX32 is down-regulated in acute lymphoblastic leukemia. Leuk Res. 2002;26(10):945-54.

132. Colley A, Beggs JD, Tollervey D, Lafontaine DL. Dhr1p, a putative DEAH-box RNA helicase, is associated with the box C+D snoRNP U3. Mol Cell Biol. 2000;20(19):7238-46.

133. Zhang Y, You J, Wang X, Weber J. The DHX33 RNA Helicase Promotes mRNA Translation Initiation. Mol Cell Biol. 2015;35(17):2918-31.

134. Liu Y, Lu N, Yuan B, Weng L, Wang F, Liu YJ, et al. The interaction between the helicase DHX33 and IPS-1 as a novel pathway to sense double-stranded RNA and RNA viruses in myeloid dendritic cells. Cell Mol Immunol. 2014;11(1):49-57.

135. Chakrabarti A, Banerjee S, Franchi L, Loo YM, Gale M, Jr., Nunez G, et al. RNase L activates the NLRP3 inflammasome during viral infections. Cell Host Microbe. 2015;17(4):466-77.

136. Hug N, Caceres JF. The RNA helicase DHX34 activates NMD by promoting a transition from the surveillance to the decay-inducing complex. Cell Rep. 2014;8(6):1845-56.

137. Melero R, Hug N, Lopez-Perrote A, Yamashita A, Caceres JF, Llorca O. The RNA helicase DHX34 functions as a scaffold for SMG1-mediated UPF1 phosphorylation. Nat Commun. 2016;7:10585.

138. Tran H, Schilling M, Wirbelauer C, Hess D, Nagamine Y. Facilitation of mRNA deadenylation and decay by the exosome-bound, DExH protein RHAU. Mol Cell. 2004;13(1):101-11.

139. Thandapani P, Song J, Gandin V, Cai Y, Rouleau SG, Garant JM, et al. Aven recognition of RNA G-quadruplexes regulates translation of the mixed lineage leukemia protooncogenes. Elife. 2015;4.

140. Booy EP, Howard R, Marushchak O, Ariyo EO, Meier M, Novakowski SK, et al. The RNA helicase RHAU (DHX36) suppresses expression of the transcription factor PITX1. Nucleic Acids Res. 2014;42(5):3346-61.

141. Bicker S, Khudayberdiev S, Weiss K, Zocher K, Baumeister S, Schratt G. The DEAH-box helicase DHX36 mediates dendritic localization of the neuronal precursor-microRNA-134. Genes Dev. 2013;27(9):991-6.

142. Chalupnikova K, Lattmann S, Selak N, Iwamoto F, Fujiki Y, Nagamine Y. Recruitment of the RNA helicase RHAU to stress granules via a unique RNA- binding domain. J Biol Chem. 2008;283(50):35186-98.

143. Sardana R, Liu X, Granneman S, Zhu J, Gill M, Papoulas O, et al. The DEAH-box helicase Dhr1 dissociates U3 from the pre-rRNA to promote formation of the central pseudoknot. PLoS Biol. 2015;13(2):e1002083.

144. Johnson SJ, Jackson RN. Ski2-like RNA helicase structures: common themes and complex assemblies. RNA Biol. 2013;10(1):33-43.

145. Halbach F, Reichelt P, Rode M, Conti E. The yeast ski complex: crystal structure and RNA channeling to the exosome complex. Cell. 2013;154(4):814-26.

146. Weir JR, Bonneau F, Hentschel J, Conti E. Structural analysis reveals the characteristic features of Mtr4, a DExH helicase involved in nuclear RNA processing and surveillance. Proc Natl Acad Sci U S A. 2010;107(27):12139-44.

147. Tremblay N, Baril M, Chatel-Chaix L, Es-Saad S, Park AY, Koenekoop RK, et al. Spliceosome SNRNP200 Promotes Viral RNA Sensing and IRF3 Activation of Antiviral Response. PLoS Pathog. 2016;12(7):e1005772.

148. Oshiumi H, Mifsud EJ, Daito T. Links between recognition and degradation of cytoplasmic viral RNA in innate immune response. Rev Med Virol. 2016;26(2):90-101.

149. Ullah R, Li J, Fang P, Xiao S, Fang L. DEAD/H-box helicases:Anti-viral and pro-viral roles during infections. Virus Res. 2022;309:198658.

150. Dhir A, Dhir S, Borowski LS, Jimenez L, Teitell M, Rotig A, et al. Mitochondrial double-stranded RNA triggers antiviral signalling in humans. Nature. 2018;560(7717):238-42.

151. Clemente P, Pajak A, Laine I, Wibom R, Wedell A, Freyer C, et al. SUV3 helicase is required for correct processing of mitochondrial transcripts. Nucleic Acids Res. 2015;43(15):7398-413.

152. Liu Z, Wang J, Cheng H, Ke X, Sun L, Zhang QC, et al. Cryo-EM Structure of Human Dicer and Its Complexes with a Pre-miRNA Substrate. Cell. 2018;173(6):1549-50.

153. Teijeiro V, Yang D, Majumdar S, Gonzalez F, Rickert RW, Xu C, et al. DICER1 Is Essential for Self-Renewal of Human Embryonic Stem Cells. Stem Cell Reports. 2018;11(3):616-25.

154. Han Q, Chen G, Wang J, Jee D, Li WX, Lai EC, et al. Mechanism and Function of Antiviral RNA Interference in Mice. mBio. 2020;11(4).

155. Dixit E, Kagan JC. Intracellular pathogen detection by RIG-I-like receptors. Adv Immunol. 2013;117:99-125.

156. Esser-Nobis K, Hatfield LD, Gale M, Jr. Spatiotemporal dynamics of innate immune signaling via RIG-I-like receptors. Proc Natl Acad Sci U S A. 2020;117(27):15778-88.

157. Franks TM, Singh G, Lykke-Andersen J. Upf1 ATPase-dependent mRNP disassembly is required for completion of nonsense- mediated mRNA decay. Cell. 2010;143(6):938-50.

158. Hogg JR, Goff SP. Upf1 senses 3'UTR length to potentiate mRNA decay. Cell. 2010;143(3):379-89.

159. Huang F, Zhang J, Zhang Y, Geng G, Liang J, Li Y, et al. RNA helicase MOV10 functions as a co-factor of HIV-1 Rev to facilitate Rev/RRE-dependent nuclear export of viral mRNAs. Virology. 2015;486:15-26.

160. Kute PM, Ramakrishna S, Neelagandan N, Chattarji S, Muddashetty RS. NMDAR mediated translation at the synapse is regulated by MOV10 and FMRP. Mol Brain. 2019;12(1):65.

161. Kenny PJ, Kim M, Skariah G, Nielsen J, Lannom MC, Ceman S. The FMRP-MOV10 complex: a translational regulatory switch modulated by G- Quadruplexes. Nucleic Acids Res. 2020;48(2):862-78.

162. Gregersen LH, Schueler M, Munschauer M, Mastrobuoni G, Chen W, Kempa S, et al. MOV10 Is a 5' to 3' RNA helicase contributing to UPF1 mRNA target degradation by translocation along 3' UTRs. Mol Cell. 2014;54(4):573-85.

163. Kenny PJ, Zhou H, Kim M, Skariah G, Khetani RS, Drnevich J, et al. MOV10 and FMRP regulate AGO2 association with microRNA recognition elements. Cell Rep. 2014;9(5):1729-41.

164. Banerjee S, Neveu P, Kosik KS. A coordinated local translational control point at the synapse involving relief from silencing and MOV10 degradation. Neuron. 2009;64(6):871-84.

165. Cuevas RA, Ghosh A, Wallerath C, Hornung V, Coyne CB, Sarkar SN. MOV10 Provides Antiviral Activity against RNA Viruses by Enhancing RIG-I-MAVS- Independent IFN Induction. J Immunol. 2016;196(9):3877-86.

166. Zhang X, Yu L, Ye S, Xie J, Huang X, Zheng K, et al. MOV10L1 Binds RNA G-Quadruplex in a Structure-Specific Manner and Resolves It More Efficiently Than MOV10. iScience. 2019;17:36-48.

167. Vourekas A, Zheng K, Fu Q, Maragkakis M, Alexiou P, Ma J, et al. The RNA helicase MOV10L1 binds piRNA precursors to initiate piRNA processing. Genes Dev. 2015;29(6):617-29.

168. Hirose T, Ideue T, Nagai M, Hagiwara M, Shu MD, Steitz JA. A spliceosomal intron binding protein, IBP160, links position-dependent assembly of intron- encoded box C/D snoRNP to pre-mRNA splicing. Mol Cell. 2006;23(5):673-84.

169. Lim SC, Bowler MW, Lai TF, Song H. The Ighmbp2 helicase structure reveals the molecular basis for disease-causing mutations in DMSA1. Nucleic Acids Res. 2012;40(21):11009-22.

170. Wagschal A, Rousset E, Basavarajaiah P, Contreras X, Harwig A, Laurent-Chabalier S, et al. Microprocessor, Setx, Xrn2, and Rrp6 co-operate to induce premature termination of transcription by RNAPII. Cell. 2012;150(6):1147-57.

171. Miller MS, Rialdi A, Ho JS, Tilove M, Martinez-Gil L, Moshkina NP, et al. Senataxin suppresses the antiviral transcriptional response and controls viral biogenesis. Nat Immunol. 2015;16(5):485-94.
